# Proliferation of activated hepatic stellate cells requires REST

**DOI:** 10.1186/s10020-025-01406-z

**Published:** 2026-01-28

**Authors:** Vladimir S Shavva, L. Tarnawski, W. Dai, N. Moruzzi, A.-S. Haller, F. Borg, S. Hansson, Q. Guo, M. Cai, E. Fekete, J.J. Vacquié, A. Maestri, T. Liu, R.S. Vimaladithan, S.G. Malin, P. Saliba-Gustafsson, P.-O. Berggren, C.E. Hagberg, O. Ahmed, Peder S. Olofsson

**Affiliations:** 1 Laboratory of Immunobiology, Center for Bioelectronic Medicine, Department of Medicine, Karolinska Institutet, Solna, Stockholm, Sweden; 2 Division of Cardiovascular Medicine, Department of Medicine, Center for Molecular Medicine, Karolinska Institutet, Karolinska University Hospital, Solna, Stockholm, Sweden; 3 Department of Molecular Medicine and Surgery, The Rolf Luft Research Center for Diabetes and Endocrinology, Karolinska Institutet, Stockholm, Sweden; 4 Department of Chemistry and Applied Biosciences, Institute of Pharmaceutical Sciences, ETH Zurich, Zurich, Switzerland; 5 Cardio Metabolic Unit, Department of Laboratory Medicine, Department of Medicine, Karolinska Institutet, Huddinge, Stockholm, Sweden; 6 Medicine Unit of Endocrinology, Theme Inflammation and Ageing, Karolinska University Hospital, Stockholm, Sweden; 7 Department of Biochemistry, College of Medicine and Medical Sciences, Arabian Gulf University, Manama, Kingdom of Bahrain; 8 Institutes of Bioelectronic Medicine, The Feinstein Institutes for Medical Research, Manhasset, NY USA

**Keywords:** Hepatic stellate cell activation, REST, Proliferation, Mitochondrial metabolism.

## Abstract

**Background:**

Activation of hepatic stellate cells (HSCs) is key in liver regeneration and the pathogenesis of chronic liver diseases, such as metabolic dysfunction-associated steatohepatitis (MASH). Activated HSCs promote liver inflammation and fibrosis which can lead to the development of liver cancer. Targeted removal of activated HSCs has shown promise in preventing liver fibrosis and liver cancer in mouse models. HSC activation is characterized by increased mitochondrial metabolism and upregulation of pro-fibrotic genes, but the underlying regulatory mechanisms remain incompletely understood. Since RE1-silencing transcription factor (REST) is known to regulate cell fate and metabolism, we investigated its involvement in HSC activation.

**Methods:**

REST-dependent mechanisms of HSC activation were studied using siRNA-mediated *REST* knock down in primary human HSCs and human HSC-like LX2 cells.

**Results:**

Knock down of *REST* in primary human HSCs and HSC-like LX2 cells reduced proliferation, promoted lipid accumulation and autophagy, and impaired mitochondrial metabolism, resulting in reduced growth. The effects were linked with REST-dependent regulation of the PI3K/AKT/mTORC1 pathway.

**Conclusions:**

Our findings identify a REST-dependent mechanism of HSC activation that is important for their activation and proliferation.

**Supplementary Information:**

The online version contains supplementary material available at 10.1186/s10020-025-01406-z.

## Background

Hepatic stellate cells (HSCs) play a key role in liver homeostasis and pathophysiology (Tsuchida and Friedman 2017; Liu et al. 2013; Sugimoto et al. 2025; Hansen et al. 2025; Kalinichenko et al. 2003; Bonnardel et al. 2019). In the healthy liver, quiescent HSCs reside within the space of Disse and orchestrate the local microenvironment by supporting vascular function, hepatocytes, and Kupffer cells, and contribute to the regulation of the metabolic zonation in the liver (Reynaert et al. 2008). When liver homeostasis is disturbed, quiescent HSCs undergo activation. This process involves a dramatic shift in HSC metabolism and gene expression, including induction of proliferation and transdifferentiation into myofibroblasts (Tsuchida and Friedman 2017; Liu et al. 2013; Sugimoto et al. 2025; Hansen et al. 2025; Kalinichenko et al. 2003; Bonnardel et al. 2019). Activated HSCs produce growth factors, cytokines and collagens, promoting inflammation and extracellular matrix remodeling (Tsuchida and Friedman 2017; Liu et al. 2013; Kalinichenko et al. 2003; Kitto and Henderson 2021; Mederacke et al. 2013; Filliol et al. 2022). Short-term activation of HSCs is critical for liver regeneration, as depletion of HSCs impairs this process (Kalinichenko et al. 2003; Kitto and Henderson 2021). However, when chronic disruption of liver homeostasis causes sustained activation of HSCs, pro-inflammatory and pro-fibrotic effects of HSC activation exacerbate liver injury, promote development of sustained liver fibrosis and, eventually, hepatocellular carcinoma (Tsuchida and Friedman 2017; Liu et al. 2013; Filliol et al. 2022). Accordingly, depletion of activated HSCs in mouse models of MASH and liver cancer abrogated disease progression (Filliol et al. 2022; Kisseleva et al. 2012; Krizhanovsky et al. 2008; Amor et al. 2020). Together, these observations support the notion that HSC activation is a key process in liver pathophysiology. A precise understanding of the mechanisms driving HSC activation could unlock new therapeutic avenues for metabolic-associated liver disease, liver cancer, and liver failure. Despite its clinical significance, the regulatory processes underlying HSC activation remain incompletely elucidated.

HSCs are characterized by expression of neuron-associated genes such as synaptophysin (SYP) (Cassiman et al. 1999; Oben et al. 2003, 2004), yet the mechanisms that regulate the neuron-associated functions in HSCs are largely unknown. Interestingly, the Repressor Element 1-silencing transcription factor (REST) is known to silence neuron-associated genes in non-neuronal cells (Lunyak et al. 2002). Downregulation of *REST* in stem cells permits induction of neuron-specific genes and differentiation of progenitors into neurons (Hwang and Zukin 2018), and is required for the induction of choline acetyltransferase expression in activated T cells (Tarnawski et al. 2023). However, although *REST* expression is lower in neurons compared with somatic cells, an adequate level of REST is important for neuronal function and survival: REST dysfunction in neurons leads to cell senescence and death, and was linked to development of Alzheimer’s and Parkinson’s diseases, and to a reduced lifespan (Hwang and Zukin 2018; Lu et al. 2014; Rocchi et al. 2021; Zullo et al. 2019). These observations raise the question of whether REST regulates aspects of HSC activation.

In this study, we investigated REST in HSC activation and found that REST is vital for proliferation of activated human HSCs in vitro. *REST* knock-down affected mitochondrial metabolism and lowered the mitochondrial membrane potential, promoted autophagy, and reduced activity of the PI3K/AKT/mTORC1 cascade, ultimately abrogating growth of activated HSCs. Accordingly, REST emerges as a key factor in HSC activation.

## Methods

### Cell culture

#### Primary human hepatic stellate cells

Primary human hepatic stellate cells were purchased from LifeNet Health (USA), thawed, seeded at the density of 5000 cells/cm^2^, and cultured according to manufacturer’s instructions. Cells were transfected with scrambled siRNA (ThermoFisher, #4390843, Waltham, Massachusetts, USA) or siRNA targeting REST (Santa Cruz, #sc-38129, Dallas, Texas, USA) using reverse transfection with RNAiMAX (ThermoFisher, #13778-150). Complexes were prepared in Opti-MEM (ThermoFisher, #31985-047) and placed in culture plates. Cells were diluted in DMEM (ThermoFisher, #31966021) with GlutaMax and 20% FBS (ThermoFisher, #10270-106), placed into the wells containing complexes mixed. Cells were incubated in DMEM/Opti-MEM with GlutaMax and 10% FBS for 24 h. Next, media was replaced with DMEM containing GlutaMax and 10% FBS. Cells were cultivated for 5 days, counted, passaged and re-transfected using the identical protocol.

#### Immortalized human hepatic stellate cells (LX2)

LX2 cells were maintained in DMEM with 10% FBS and seeded at the density of 21,000 cells/cm^2^. Cells were transfected with scrambled siRNA (ThermoFisher, #4390843), siRNA targeting REST (Santa Cruz, #sc-38129), siRNA targeting phosphatidylinositol 3-kinase regulatory subunit α (PIK3R1) (ThermoFisher, #AM6708, assay ID 118085) or siRNA targeting regulatory-associated protein of mTOR (RAPTOR) (ThermoFisher, #AM6708, assay ID 140660) using reverse transfection as described above. After 24 h, media was replaced with DMEM containing GlutaMax and 10% FBS or DMEM with GlutaMax and without FBS for experiments involving serum starvation. For experiments involving chemical inhibitors, cells were exposed to inhibitors 24 h after transfection and incubated for 72 h. For experiments involving rapamycin, cells were exposed to the inhibitor for 1 h and media was replaced with fresh DMEM with 10% FBS. For all experiments, cells were harvested 96 h after transfection.

#### Generation of a stable LX2 cell line with an activation reporter gene

The reporter construct indicating LX2 activation was a kind gift from Drs. Li and Xiang at the Peking University Health Science Center. Briefly, the COL1A1 promoter sequence was used to regulate the expression of the enhanced green fluorescent protein (EGFP) (pLVX-COL1A1-EGFP). The COL1A1 promoter was amplified, and the EF-1α promoter of plasmid pWPI was replaced with the COL1A1 promoter to obtain the recombinant plasmid pWPI-COL1A1. The internal ribosome entry site (IRES) sequence before the EGFP sequence of pWPI-COL1A1 was replaced with a Kozak sequence (GCCACCATGG) to obtain the recombinant plasmid pWPI-COL1A1-EGFP. The fragment consisting of the COL1A1 promoter, Kozak sequence, and EGFP was amplified, and replaced the CMV promoter and AcGFP1 on the pWVX-AcGFP1-N1 plasmid, resulting in the final recombinant plasmid pLVX-COL1A1-EGFP. Lentiviral vectors containing the LX2-activation reporter were packed in HEK293T cells using the pCMVdR8.91 and pMD2.G packaging plasmids according to standard protocols. LX2 cells were transduced using the lentiviral vectors overnight, and transduced cells were selected using 2.5 µg/mL puromycin for two days. For maintenance and subculturing, cell culture media was supplemented with 0.5 µg/mL puromycin, which was withdrawn prior to experimentation.

### IncuCyte

Primary human hepatic stellate cells were thawed, seeded, transfected and cultured as described above. At day 6 post seeding, cells were trypsinized, counted and seeded on 96-well plates at the density of 1600 cells/well in DMEM + GlutaMax + 10% FBS. After 48 h media was replaced with fresh DMEM + GlutaMax + 10% FBS or DMEM + GlutaMax, and cells were placed in IncuCyte (Sartorius, Germany).

LX2 cells were seeded in 96-well plate at the density of 3000 cells/cm^2^ and transfected as described above. 24 h after transfection, media was replaced with fresh DMEM + GlutaMax + 10% FBS, and cells were placed in IncuCyte (Sartorius). For experiments involving chemical inhibitors, inhibitors were added to the media 24 h after transfection.

Cells were photographed every 4 h for 72 h. Analysis of confluence was performed with Incucyte 2022B Rev2 software using phase channel, AI confluence segmentation. For area under the curve (AUC) calculation, confluence was normalized to confluence at time point 0 per well. Experiments were repeated 1–4 times, with 3–6 biological replicates per experiment.

### RNA isolation, reverse transcription and quantitative polymerase chain reaction (RT-qPCR)

RNA from primary human hepatic stellate cells was isolated using RNeasy Mini Kit (Qiagene, #74104, Germany). RNA from LX2 cells was isolated using QIAzole (Qiagene, #79306). DNA digestion was performed using RNase-Free DNase (Qiagene, #79254). RNA concentration was measured using NanoDrop 2000 (ThermoFisher). 0.1 to 1 µg of RNA was used for RT. RT was performed using High-Capacity cDNA Reverse Transcription Kit (ThermoFisher, #4368814). qPCR was performed in QuantStudio 7 Pro (ThermoFisher) using GoTaq Probe qPCR kit (Promega, #A6102, Madison, Wisconsin, USA) for Taqman qPCR or SsoAdvanced Universal SYBR Green Supermix (Bio-Rad, #1725274, Hercules, California, USA) for SYBR Green qPCR and primers listed in Table S1. mRNA levels were calculated as 2^Ct(control)−Ct(sample)^ and normalized to geometric mean of housekeeping genes mRNA level (*RPLP0*, *PPIA*, *SDHA*). Experiments were repeated 2–4 times, with 3 biological replicates per experiment.

### SeaHorse extracellular flux analysis

Metabolic analysis was performed using the Seahorse XF964 Flux Analyzer (Agilent, Santa Clara, CA, U.S.). LX2 cells were transfected with scrambled siRNA or siRNA targeting REST as described. 24 h before SeaHorse analysis LX2 cells were re-seeded in 96-well XF Cell Culture Microplates (40000 cells/well) in complete DMEM medium with 10% FBS and GlutaMax. Efficiency of *REST* KD and expression of key markers following re-plating is shown in Fig. S2d. 1 h prior to the analysis the medium was replaced with XF medium containing 5.5 mM glucose, 2 mM GlutaMax (ThermoFisher), 1 mM sodium pyruvate. Cells were incubated at 37 °C without CO_2_ for 1 h prior to the experiment. The oxygen consumption rate (OCR) was recorded at basal level and following injection of oligomycin (1 μM final), carbonyl-cyanide 4-trifluoromethoxy-phenylhydrazone FCCP (0.5 + 0.5 µM), and mixture of rotenone and antimycin A (5 µM). Running template was 2´ mix and 4,5´ measure. All chemicals were purchased from Sigma-Aldrich. Data were normalized on the number of cells per well. Normalization for cell number was carried out staining the nuclei with Hoechst 33,342 (Molecular Probes, Eugine, Oregon, USA) for 10´ and then imaging each well using BD pathway 855 (BD Biosciences, Franklin Lakes, U.S.) with 10x objective and montage 5 × 4. Cell number was counted with Cell profiler software. Experiments were repeated 2 times, with 11–12 biological replicates per experiment.

### Western blotting & ELISA

LX2 cells were lysed in RIPA buffer with protease/phosphatase inhibitors (ThermoFisher, #1861281) (10 min, 4 °C). Protein concentration was determined using DC protein assay (Bio-Rad, #5000111) or Bradford assay (Bio-Rad, #500 − 0205). Proteins were segregated in 8–15% polyacrylamide gels and transferred onto PVDF membranes using wet transfer. Protein loading was determined using Ponceau Red S staining. Membranes were blocked with 5% blocking reagent (Cell Signaling, #9999S, Denver, Massachusetts, USA) or 5% BSA diluted in PBS/0.05% Tween20, stained with primary (Table S2) and secondary (Table S3) antibodies, and secondary antibody fluorescence was measured using ChemiDoc (Bio-Rad). Alternatively, protein extracts were analyzed using Simple Jess capillary system using anti-rabbit and anti-mouse secondary antibody (Biotechne, #DM-001) and Jess capillary plates (Biotechne, #SM-W004). Original images for westerns blots are shown in Fig. S5 and S6. Experiments were repeated 2–6 times, with 1 or 3 biological replicates per experiment.

COL1A1 concentration in media was measured using ELISA (R&D Systems, #DY6220-05) according to manufacturers instructions.

### Flow cytometry

Primary HSCs were cultured as described above. Following trypsinization, cells were washed in PBS -/- with 10% FBS. Prior to antibody staining, cells were incubated in the blocking buffer containing PBS -/- and 10% FBS. Cells were stained using (a) LipidTox Red (ThermoFisher, #H34476) and 1 µM monodansylcadaverine (Sigma, #D4008) or (b) 50 nM MitoTracker Deep Red FM (ThermoFisher, #M22426) and 10 µg/mL JC-1 (ThermoFisher, #T3168) in PBS -/- for 30 minutes at 4 C (a) or room temperature (b). Viability was assessed using Zombie Yellow™ Fixable Viability Dye (BioLegend, #423102) or 1 µg/mL DAPI. For detection of ROS, cells were stained with 2’,7’-dichlorofluorescin diacetate (Millipore, #287810, Burlington, Massachusetts, USA) for 30 min. at 37 °C, washed with HBSS and fixed with 10% methanol/HBSS for 5 min. at room temperature. The gating strategies for flow cytometry are shown in Figures S7 & S8. Stained cells were analyzed using the Cytek Aurora 5 laser system (Cytek) and FlowJo software v.10 (FlowJo). Experiments were repeated 2–6 times, with 3 biological replicates per experiment.

### Immunocytochemistry and confocal microscopy

Primary HSCs (4250 cells/cm^2^) and LX2 cells (25000 cells/cm^2^) were cultured on Ibidi 18-well µ-Slide. Cells were transfected with siRNA as described above. Cells were fixed in 2% PFA/PBS, blocked with 1% BSA in 0.1% Tween-20/PBS and stained with antibodies (Table S4) overnight at 4 °C. Cells were washed with PBS and stained with secondary antibodies for 1 h at 4 °C. Next, cells were washed with PBS, stained with DAPI (1 µg/mL) and maintained in 10% glycerol/PBS at 4 °C.

Images were acquired with a confocal laser scanning microscope (Nikon, ECLIPSE Ti2) equipped with 10x (N/A = 0.45) and 20x (N/A = 0.75) objectives and NIS-Elements Software (version 5.11.01). For quantification of COL1A1 and αSMA immunofluorescence, three to five fields of view (10x objective, 1269.76 × 1269.76 μm) were acquired per donor per condition using the same settings. Images were analyzed using QuPath software (version 0.5.1). Cell area was determined by the area above a set threshold. Total fluorescent signals of each cell were calculated by the mean fluorescent intensity of the cell times the area. Experiments with LX2 cells were conducted 1 time, with 6 biological replicates per experiment.

### CellTrace

CellTrace™ cell proliferation assay (ThermoFisher, #C34564) was performed according to the manufacturer’s instructions. Briefly, following trypsinization, LX2 cells were resuspended in the CellTrace working solution (1 µM). Cells were incubated for 20 min at 37 °C. The reaction was quenched by DMEM with 2% FBS for 5 min at room temperature. Next, 4 × 10^4^ cells per well were plated in a 24-well plate and transfected with siRNA as described above. After 24 h media was changed to DMEM + GlutaMax with 10% FBS. 96 h after plating cells were trypsinised and fixed with 1% PFA. CellTrace accumulation was measured using Cytek Northern Lights flow cytometer (Cytek Biosciences, #NL-3000, Fremont, California, USA) and analyzed using FlowJo software version (10.10.0). Experiments were repeated 2 times, with 3 biological replicates per experiment.

### Mitochondrial/Genomic DNA ratio

Total DNA was extracted using Genomic DNA GeneJET Purification Kit (ThermoFisher, #K0721). Mitochondrial and genomic DNA were quantified by qPCR using SsoAdvanced Universal SYBR Green Supermix (Bio-Rad, #1725274). Mitochondrial DNA was quantified using primers for tRNA-Leu (5-CACCCAAGAACAGGGTTTGT-3, 5-TGGCCATGGGTATGTTGTTA-3) (Rooney et al. 2015). Genomic DNA was quantified using primers for GAPDH pseudogene (5- AAGGGCATCCTGGGCTAC-3, 5-GTGGAGGAGTGGGTGTCG-3). Experiments were repeated 2 times, with 3 biological replicates per experiment.

### RNA-sequencing

Primary human HSCs were activated by plating for 12 days in DMEM containing 10% FBS and transfected with scrambled siRNA or siRNA targeting REST. Cells were exposed to fresh DMEM with 10% FBS 24 h before RNA extraction. RNA was extracted using RNeasy Mini Kit (Qiagene, #74104) with DNA digestion (Qiagene, #79254). Pair-end mRNA sequencing of poly-A enriched RNA with the depth of 90 million reads was performed at Novogene. Alignment to human transcriptome (release hg38) and quantification was performed with Salmon (Patro et al. 2017). Data was imported (Soneson et al. 2016) and differential expression of transcripts was analyzed with DESeq2 (Love et al. 2014). Heatmaps were plotted using pheatmap. Pathway analysis was performed with clusterProfiler (Wu et al. 2021).

### Statistics

Statistical analysis was performed using the R package ggpubr. Normality testing was performed using the Shapiro test. Pair-wise comparison was performed using Student’s *t* test or Mann-Whitney *U* test followed by multiple comparison correction using Hommel test or FDR test. Statistical tests are indicated in figure legends. Dots represent independent biological replicates. The minimum of 6 biological replicates were performed for each measurement in LX2 cells. In experiments involving primary human HSCs cells isolated from 4 individual donors were used. The interquartile range method was used for outlier detection. Outliers are shown as blue dots. *P* < 0.05 was considered significant.

## Results

### *REST* regulated HSC activation

**Fig. 1:**
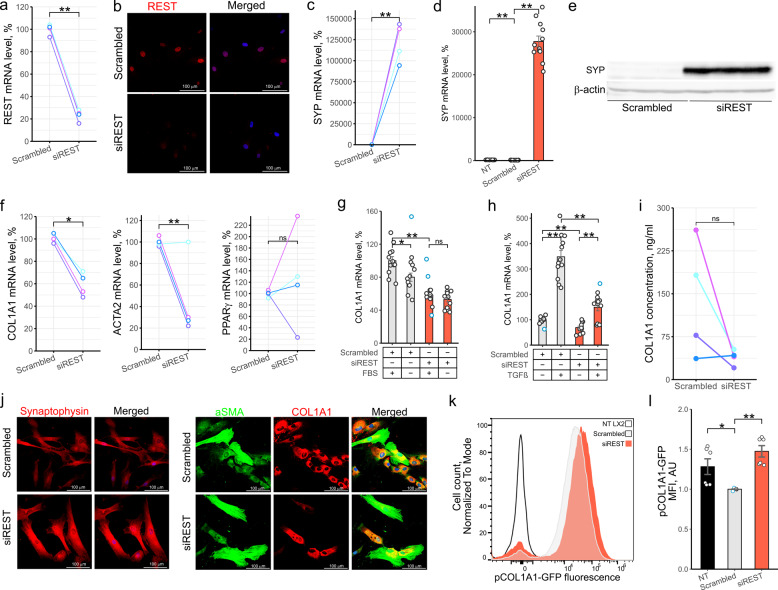
REST regulated growth of activated HSCs.** a-c**,** f**,** i**,** j** Primary human HSCs were activated by plating and transfected with scrambled siRNA or siRNA targeting *REST* for 12 days. **a**,** c**,**f** qPCR. Dots show mRNA level of genes of interest in activated phHSCs normalized to *PPIA*, *RPLP0* and *SDHA* (*n* = 4). Colors indicate HSCs from individual donors (paired *t* test). **b** Confocal microscopy. Representative images of REST protein level in activated phHSC (*n* = 4). **d** qPCR. *SYP* expression in LX2 cells following 96 h transfection with scrambled siRNA or siRNA targeting *REST* (*n* = 12). Bars show mean ± SEM, normalized to *PPIA*, *SDHA* and *RPLP0* and expressed relative to mean of scrambled siRNA-transfected cells (*t* test, Hommel test for multiple comparisons correction). **e** Western blotting. SYP protein level in LX2 cells following 96 h transfection with scrambled siRNA or siRNA targeting *REST* (*n* = 6). **g** qPCR. *COL1A1* expression in LX2 cells following 96 h transfection with scrambled siRNA or siRNA targeting *REST*. Cells were exposed to media containing 10% or 0% FBS for 72 h (*n* = 11). Bars show mean ± SEM, normalized to *PPIA*, *SDHA* and *RPLP0* and expressed relative to mean of scrambled siRNA-transfected cells (*t* test, Hommel test for multiple comparisons correction). **h** qPCR. *COL1A1* expression in LX2 cells following 96 h transfection with scrambled siRNA or siRNA targeting *REST*. 24 h after transfection, cells were serum starved for 24 h and exposed to 5 ng/mL of TGFβ for 48 h (*n* = 12). Bars show mean ± SEM, normalized to *PPIA*, *SDHA* and *RPLP0* and expressed relative to mean of scrambled siRNA-transfected cells (*t* test, Hommel test for multiple comparisons correction). **i** Quantification of COL1A1 protein secretion from activated phHSCs by ELISA (*n* = 4). Dots show REST protein level. Colors indicate HSCs from individual donors (paired *t* test). **j** Confocal microscopy. Representative images of COL1A1, αSMA and SYP protein level in phHSCs (*n* = 4). Merged – images showing nuclear staining (DAPI) and protein stainings. **k-l** Flow cytometry. LX2 carrying EGFP under control of COL1A1 promoter were transfected with scrambled siRNA or siRNA targeting *REST* for 96 h. NT LX2 – non-transfected cells. **k** Representative histogram of GFP fluorescence (*n* = 6). y axis shows cell count normalized to mode. **l** Bar graph showing GFP fluorescence (*n* = 6). The Y axis shows MFI (mean fluorescence intensity) normalized to scrambled siRNA-transfected cells (Mann-Whitney *U* test, Hommel test for multiple comparisons correction). NT – non-transfected. Outliers are indicated in blue. *p < 0.05, **p < 0.01

To study the function of *REST* in HSC activation, we activated primary human HSCs (phHSCs) for 12 days and exposed cultures to siRNA targeting *REST* (siREST) or scrambled siRNA throughout the activation period. qPCR and immunofluorescence showed significant *REST* knock-down (KD) by siREST (Fig. 1a, b). *REST* KD strongly upregulated the transcript levels of synaptophysin (*SYP*), a direct target inhibited by REST (Lietz et al. 2003) (Fig. 1c). Similarly, *REST* KD in the human hepatic stellate cell line LX2 (Xu et al. 2005) elevated *SYP* (Fig. 1d, e & Fig. S1a, b). Together, this shows a functional KD of *REST* in HSCs.

Next, we assessed the role of REST in activated HSCs. *REST* KD significantly downregulated pro-fibrogenic collagen 1α1 (*COL1A1*) and the activated HSC marker α smooth muscle actin (*ACTA2*), but did not significantly affect quiescence marker peroxisome proliferator-activated receptor γ (PPARγ) (Tsuchida and Friedman 2017) (Fig. 1f). Similarly, *REST* KD in the human hepatic stellate cell line LX2 reduced *COL1A1* (Fig. S1c). Further, both *REST* KD and inhibition of activation using 72 h serum starvation reduced transcript levels of *COL1A1* in LX2 cells (Fig. 1g, Fig. S1d). Of note, serum starvation did not further reduce *COL1A1* levels beyond *REST* KD, and *REST* mRNA levels were higher after serum starvation (Fig. S1d, e), indicating that serum starvation downregulated *COL1A1* by a mechanism that requires REST (Fig. 1g). Thus, *REST* KD reduced the mRNA levels of the key pro-fibrogenic gene *COL1A1* in activated HSCs.

We next investigated the role of REST in the response of activated HSCs to re-exposure to pro-activation microenvironments. In activated phHSCs exposed to fresh FBS (10%), *REST KD* promoted a significant upregulation of *ACTA2* and *SYP* transcripts but caused no significant change in the mRNA level of *COL1A1* (Fig. S1f). Similarly, in cultures of activated LX2 cells, *REST* KD did not abrogate the TGFβ-dependent induction of *COL1A1* (Fig. 1h, Fig. S1g). Of note, TGFβ exposure did not significantly alter *REST* transcript levels (Fig. S1g, h) and *REST* KD reduced the relative level of *COL1A1* (Fig. 1h), corroborating REST-dependent regulation of *COL1A1*. Together, these data indicate that although *REST* KD reduces *COL1A1* expression, re-exposure of activated HSCs to a pro-activation microenvironment can upregulate *COL1A1*.

Next, we asked whether levels of COL1A1 protein are regulated by *REST* in activated HSCs. *REST* KD reduced secretion of COL1A1 from activated phHSCs in cell cultures from three out of four donors (Fig. 1i), suggesting that *REST* promotes activated HSC-mediated fibrogenesis. Surprisingly, intracellular COL1A1 protein levels were increased after *REST* KD in both phHSCs (Fig. 1j, Fig. S1j) and LX2s (Fig. S1i) as measured by confocal microscopy immunofluorescence. To test this further, we created the pLVX-LX2 cell line (Fig. S1k) where GFP expression is controlled by COL1A1 promoter. *REST* KD in pLVX-LX2 cells lead to a reduction in *COL1A1* mRNA levels (Fig. S1l), but increased GFP fluorescence (Fig. 1k, l, Fig. S1m). Together, these data suggest that *REST* KD regulates synthesis and secretion of COL1A1 independently, and induced accumulation of intracellular COL1A1 protein. Collectively, these results indicate that *REST* regulates the in vitro pro-fibrogenic activity of activated HSCs.

### *REST* regulated HSC proliferation and survival

**Fig. 2:**
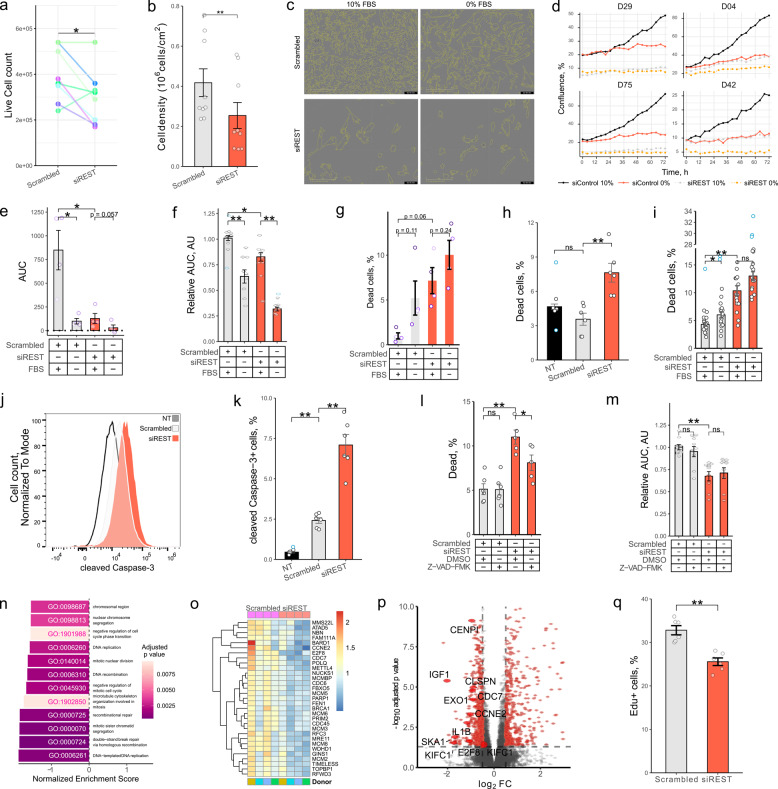
REST regulates survival and proliferation of HSCs.** a** Primary human HSCs were activated by plating and transfected with scrambled siRNA or siRNA targeting *REST* for 6 days (*n* = 8). Live cell counts were quantified using trypan blue staining (paired *t* test). **b** LX2 cells were transfected with scrambled siRNA or siRNA targeting REST for 96 h (*n* = 9). Live cell counts were quantified using trypan blue staining (*t* test). **c-e** Analysis of phHSC growth using IncuCyte (*n* = 4). PhHSCs were transfected with scrambled siRNA or siRNA targeting *REST*. After 6 days cells were passaged, re-transfected and incubated with 10% FBS for 48 h. Next, cells were placed into IncuCyte and media was changed to DMEM or DMEM + 10% FBS and photographed for 72 h every 4 h. **c** Representative images of primary human HSCs from D29 following 12 d of activation and siRNA transfection. Yellow shows cell borders. **d** Change in confluence. Y axis shows confluence (%), and x axis shows time, donor ID indicated on top. **e** Area under the curve (AUC). Confluence was normalized to confluence at day 0 per well. Bars show mean AUC ± SEM (Mann-Whitney *U* test, FDR multiple comparison correction test). **f**,** m** Bar graph of relative LX2 growth (f: *n* = 9, m: *n* = 9) presented in Fig. S2b. LX2s were transfected with siRNA for 24 h and incubated in IncuCyte for 72 h. Photographs were taken every 4 h. Y axis shows area under the curve (AUC) normalized to mean of scrambled siRNA exposed to 10% FBS (Mann-Whitney *U* test, Hommel test for multiple comparison correction). **g** Bar graph of phHSC viability following activation and *REST* KD (*n* = 4). Dead cells were quantified using zombie yellow staining and flow cytometry. Bars show mean ± SEM % of dead cells from single cell gate, dots indicate individual donors (paired *t* test with FDR test for multiple comparisons correction). **h**,** i**,** l** Bar graph of LX2 viability after *REST* KD (h, l: *n* = 6, i: *n* = 18). Dead cells were quantified by propidium iodide (i) or zombie yellow (h, i, l) staining and flow cytometry. Bars show mean ± SEM % of dead cells from single cell gate (Mann-Whitney *U* test, Hommel test for multiple comparisons correction). **j**,** k** Flow cytometry. Representative histogram (j) and bar graph (k) of cleaved Caspase-3 fluorescence (*n* = 6). Bars show mean ± SEM % of cells from single cell gate (Mann-Whitney U test, Hommel test for multiple comparisons correction). **l**,** m** LX2 cells were transfected with scrambled siRNA or siRNA targeting *REST* for 24 h and exposed to DMSO (vehicle) or Z-VAD-FMK (10 µM) for 72 h. **n-o** RNA-sequencing analysis of phHSC transcriptomes following 12 d activation and transfection (*n* = 4). Cells were exposed to fresh DMEM with 10% FBS for 24 h before harvesting. **n** Bar graph of normalized enrichment scores (NES) of GO terms related to proliferation. Gene Set Enrichment Analysis (GSEA) was performed with *p* value cutoff of 0.05. Color indicates adjusted p value. **o** Heatmap of transcripts per million (TPM) for genes belonging to GO:0006261 “DNA-templated DNA replication”. TPMs were normalized to average of TPMs per gene. **p** Volcano plot of top 10 most affected genes belonging to proliferation pathways shown in Fig. 2n. Horizontal dashed line indicates *p* value cutoff of 0.05, vertical dashed line indicates log_2_ fold change cutoff of 0.5. **q** Bar graph of LX2 proliferation measure by EdU staining and flow cytometry (*n* = 6) (*t* test with Hommel test for multiple comparisons correction). NT – non-transfected. Outliers are indicated in blue. *p < 0.05, **p < 0.01

Interestingly, we found that cell numbers in phHSC and LX2 cell cultures were significantly lower following *REST* KD as compared with exposure to scrambled siRNA (Fig. 2a, b). To investigate this, we used an IncuCyte^®^ live cell imaging system to measure HSC growth with and without concomitant serum starvation. We knocked down *REST* in phHSCs during activation for nine days and subsequently cultured the cells in the IncuCyte^®^ live cell imaging system for an additional three days in media supplemented with 10% or 0% FBS. Subsequent analysis revealed that *REST* KD reduced the confluence of activated HSC to a similar extent as did serum starvation (Fig. 2c-e). Similarly, *REST* KD reduced growth of LX2 cells assessed using IncuCyte (Fig. 2f, Fig. S2a, b).

REST was observed to promote neuronal survival leading us to hypothesize that REST KD reduces growth of activated HSCs by inducing programmed cell death. *REST* KD promoted phHSC death with an effect size similar to that of serum starvation (Fig. 2g), and in LX2 cells to an even higher extent than serum starvation (Fig. 2h, i). To study the mechanism, we next asked if *REST* KD promotes HSC apoptosis. *REST* KD increased Caspase-3 cleavage in LX2 cells (Fig. 2j-k), and exposure of LX2 cells to the caspase inhibitor Z-VAD-FMK attenuated *REST* KD-mediated cell death (Fig. 2l), identifying a role for apoptosis in *REST* KD-driven cell death. In contrast, exposure of LX2 cultures to Z-VAD-FMK did not significantly impact cell growth (Fig. 2m) with or without *REST* KD, indicating that inhibition of *REST* KD-driven apoptosis is not sufficient to restore growth of activated HSCs, and that other mechanisms control REST-dependent growth.

Accordingly, we proceeded to investigate whether *REST* KD controls HSC proliferation. First, activated phHSCs with and without *REST* KD were studied using RNA-sequencing and gene set enrichment analysis (GSEA). Proliferation-related GO terms were selected from the identified pool of significantly regulated gene sets. We observed that pathways linked to proliferation were downregulated in phHSCs after *REST* KD (Fig. 2n). To gain more insight, we asked how *REST* KD affected mRNA levels of genes included in the gene set with the lowest normalized enrichment score among these pathways, i.e. GO:0006261 “DNA-templated DNA replication”. Analysis across four independent donor cultures showed that *REST* KD consistently reduced transcript levels across the GO:0006261 gene set (Fig. 2o). Further, the top 10 most differentially expressed transcripts from the GO sets related to proliferation (Fig. 2n) were similarly downregulated following *REST* KD (Fig. 2p). Further, the frequency of EdU + cells following *REST* KD in LX2 cells was reduced, indicating lower proliferation (Fig. 2q). *REST* KD had significantly higher levels of CellTrace fluorescence intensity after 96 h of culture as compared with LX2 cells exposed to scrambled siRNA (Fig. S2c), indicating reduced cell division. Together, these results show that REST regulates proliferation of activated HSCs.

### *REST* regulated metabolism in activated HSCs

**Fig. 3:**
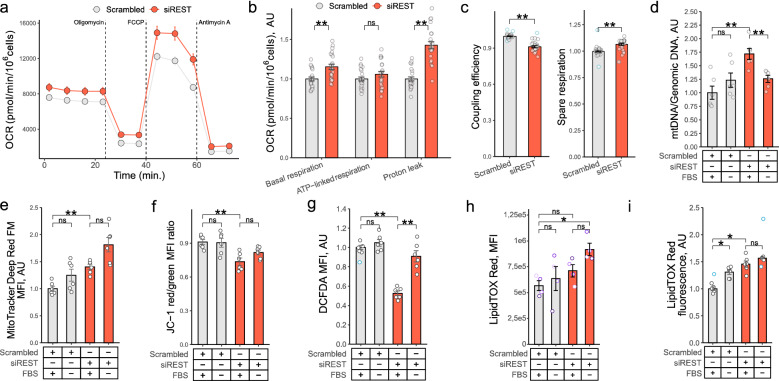
REST regulates mitochondrial metabolism of activated HSCs.** a-g**,** i** LX2 cells were transfected with scrambled siRNA or siRNA targeting REST. 24 h after transfection, cells were exposed to fresh medium containing DMEM with 10% FBS or 0% FBS for 72 h. **a** Oxygen consumption rate (OCR) was measured in LX2 cells using SeaHorse. X axis shows time, y axis shows OCR (*n* = 23). **b-c** Bar graph showing OCR in LX2 cells in arbitrary units, AU (*n* = 23). Samples were normalized to LX2 cells transfected with scrambled siRNA (Mann-Whitney *U* test, Hommel test for multiple comparison correction). **d** Bar graphs of mitochondrial to genomic DNA ratio in LX2 cells (*n* = 6). PCR. Samples were normalized to LX2 cells transfected with scrambled siRNA incubated in DMEM with 10% FBS (*t* test, Hommel test for multiple comparison correction). **e** Bar graphs of MitoTracker Deep Red FM fluorescence in LX2 cells (*n* = 6). Flow cytometry. Samples were normalized to LX2 cells transfected with scrambled siRNA incubated in DMEM with 10% FBS (*t* test, Hommel test for multiple comparison correction). **f** Bar graphs of JC-1 red/green fluorescence ratio in LX2 cells (*n* = 6). Flow cytometry. Samples were normalized to LX2 cells transfected with scrambled siRNA incubated in DMEM with 10% FBS (*t* test, Hommel test for multiple comparison correction). **g** Bar graphs of DCFDA fluorescence in LX2 cells (*n* = 6). Flow cytometry. Samples were normalized to LX2 cells transfected with scrambled siRNA incubated in DMEM with 10% FBS (*t* test, Hommel test for multiple comparison correction). **h** Bar graph of LipidTOX Red fluorescence in phHSCs (*n* = 4). HSCs were activated by plating and transfected with scrambled siRNA or siRNA targeting REST for 12 days. 72 h prior to harvesting cells were exposed to fresh media containing DMEM with 10% or 0% FBS. Bars show MFI from single cell gate, and dots indicate individual donors (paired *t* test with FDR test for multiple comparisons correction). **i** Bar graph of LipidTOX Red staining of LX2s (*n* = 6). Samples were normalized to LX2 cells transfected with scrambled siRNA incubated in DMEM with 10% FBS (Mann-Whitney *U* test, Hommel test for multiple comparison correction). MFI – mean fluorescence intensity. AU – arbitrary units. Outliers are indicated in blue. *p < 0.05, **p < 0.01

Metabolic reprogramming is key for HSC activation (Tsuchida and Friedman 2017; Liu et al. 2013; Fondevila et al. 2022, 2024), and blocking oxidative phosphorylation abrogates proliferation of activated HSCs (Smith-Cortinez et al. 2020). Since REST has been linked to metabolic regulation of neuronal function (Garriga-Canut et al. 2006; Cavadas et al. 2016), we asked whether *REST* regulated proliferation of activated HSCs through control of mitochondrial function. Analysis of the oxygen consumption rate (OCR) revealed that the basal respiration was elevated in LX2 cells with *REST* KD (Fig. 3a-c). The ratio of mitochondrial to genomic DNA was higher in *REST* KD (Fig. 3d) as was MitoTracker Deep Red FM fluorescence (Fig. 3e), indicating an increase in mitochondrial mass and mitochondrial numbers. ATP-linked respiration was unchanged while proton leakage was significantly higher in LX2 cells with *REST* KD, observations that were further supported by the decrease in coupling efficiency and the increase in the spare respiratory capacity (Fig. 3c). In line with these findings, JC-1 staining (Reers et al. 1991) indicated that the mitochondrial membrane potential was lower following *REST* KD (Fig. 3f). Mitochondrial metabolism is a major source of reactive oxygen species (ROS), and mitochondria-derived ROS were recently observed to directly drive cell cycle progression (Kirova et al. 2022). Dichlorofluorescin diacetate (DCFDA) staining (Ferrer et al. 1990) indicated reduced ROS production in *REST* KD LX2 cells as compared with LX2 cells exposed to scrambled siRNA (Fig. 3g). Together, the data links *REST* with mitochondrial metabolism and growth in activated HSCs.

**Fig. 4:**
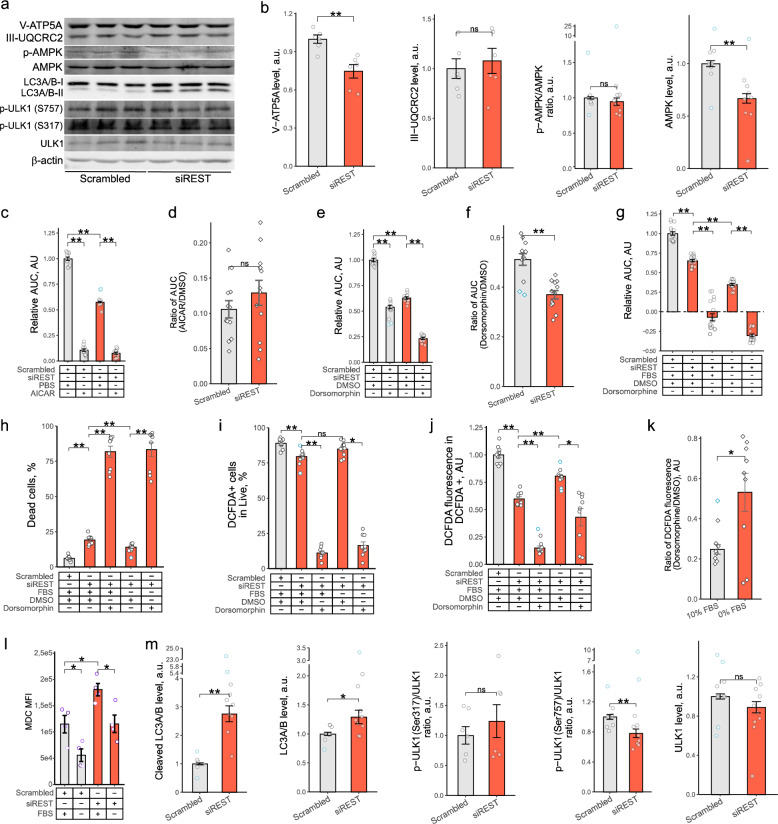
REST regulated AMPK activity and autophagy in activated HSCs.** a-k**,** m** LX2 cells were transfected with scrambled siRNA or siRNA targeting *REST*. 24 h later, cells were exposed to fresh medium containing DMEM with 10% FBS or 0% FBS and (c, e, g-k) DMSO (vehicle), AICAR (2 mM) or dorsomorphin (10 µM) for 72 h. **a-b**,** m** Western blotting of V-ATP5A (*n* = 6), III-UQCRC2 (*n* = 6), AMPK (*n* = 9), LC3A/B (*n* = 10) and ULK1 (*n* = 10) in LX2 cells. Representative images are shown. β-actin is shown as loading control. **c**,** e**, **g** Bar graph of relative LX2 growth (*n* = 12) presented in Fig. S3b, c, e. Cells were incubated in Incucyte for 72 h in DMEM with 10% FBS (c, e) or 0% FBS (g) and photographed every 4 h. Y axis shows area under the curve (AUC) normalized to mean of scrambled siRNA exposed to vehicle. Bar show mean ± SEM in arbitrary units, AU (*t* test, Hommel test for multiple comparison correction). **d**,** f** Bar graph showing ratios of *REST* siRNA-transfected samples to mean of scrambled siRNA-transfected samples (*t* test, Hommel test for multiple comparisons correction). **h** Bar graph of LX2 viability after *REST* KD (*n* = 12). Dead cells were quantified by zombie yellow staining and flow cytometry. Bars show mean ± SEM % of dead cells from single cell gate (Mann-Whitney *U* test, Hommel test for multiple comparisons correction). **i-j** Bar graphs of the frequency of DCFDA + cells (i) or DCFDA fluorescence (j) in LX2 cells (*n* = 12). Flow cytometry. Samples were normalized to LX2 cells transfected with scrambled siRNA incubated in DMEM with 10% FBS (Mann-Whitney *U* test, Hommel test for multiple comparison correction). **k** Bar graph showing ratios of DCFDA fluorescence of samples incubated in 10% FBS to mean of serum-starved samples in (j) (*t* test, Hommel test for multiple comparisons correction). **l** Bar graph of monodansylcadaverine (MDC) staining of phHSCs (*n* = 4). HSCs were activated by plating and transfected with scrambled siRNA or siRNA targeting *REST* for 12 days. 72 h prior to harvesting cells were exposed to fresh media containing DMEM with 10% or 0% FBS. Dots indicate individual donors. Bars show mean ± SEM (paired *t* test, FDR test for multiple comparison correction). Outliers are indicated in blue. *p < 0.05, **p < 0.01

Since utilization of intracellular lipid deposits and mitochondria-dependent ATP production via β-oxidation is key for HSC activation (Tsuchida and Friedman 2017; Liu et al. 2013; Fondevila et al. 2022, 2024), we next asked whether *REST* KD affected the energy balance. LipidTOX Red staining indicated that *REST* KD elevated levels of intracellular lipids in phHSCs (Fig. 3h) and LX2s (Fig. 3i, Fig. S3a), and western blotting revealed a reduction in mitochondrial ATP synthase (V-ATP5A) protein levels in LX2 cells after *REST* KD (Fig. 4a, b). These observations are congruent with reduced lipid utilization and ATP synthesis to support proliferation in *REST* KD HSCs.

Reduction of ATP production leads to an increase in AMP/ATP ratio and activation of AMP activated protein kinase (AMPK). Since induction of AMPK activity was observed to limit HSC activation (Caligiuri et al. 2008), we asked whether AMPK plays a role in REST-driven regulation of HSC growth. We did not observe consistent elevation of AMPKα Thr172 phosphorylation following *REST* KD (Fig. 4a, b). However, the total amount of AMPK was reduced following *REST* KD (Fig. 4a, b). Induction of AMPK activity using 5-Aminoimidazole-4-carboxamide ribonucleotide (AICAR) abrogated HSC growth in both scrambled- and *REST* siRNA-transfected LX2 cells (Fig. 4c-d, Fig. S3b), suggesting that AMPK was not fully activated in cell cultures. Inhibition of AMPK basal activity with dorsomorphin reduced LX2 growth, and *REST* KD further exacerbated this dorsomorphin-driven effect (Fig. 4e-f, Fig. S3c). Together, we observed that strong activation of AMPK stopped growth while inhibition of the basal AMPK activity reduced growth of HSCs, supporting the notion that basal activity of AMPK is required for cell growth. Thus, inhibition of AMPK aggravated the effects on proliferation by *REST* KD, further supporting interaction between AMPK and REST signaling in regulation of growth.

We observed that *REST* KD reduced ROS levels and increased mitochondrial to genomic DNA ratio in LX2 cells, and serum starvation abrogated these effects (Fig. 3d, g). Nutrient starvation induces AMPK activity (Herzig et al. 2018), and AMPK activity was required for LX2 growth following *REST* KD (Fig. 4e). Accordingly, we asked if AMPK activation following serum starvation in *REST* KD LX2 cultures is linked to increase in ROS production and reduction in mitochondrial biogenesis. To test this, we knock down *REST* in LX2 cells, incubated cells in DMEM with or without FBS and exposed cultures to vehicle or AMPK inhibitor dorsomorphin. Inhibition of AMPK following *REST* KD reduced growth, and this effect was exacerbated by serum starvation (Fig. 4g, Fig. S3d). Further, frequency of LX2 death was elevated in dorsomorphin-exposed cells (Fig. 4h), supporting that notion that AMPK is required for LX2 survival following *REST* KD. Frequency of cells with detectable levels of ROS was reduced in cells exposed to dorsomorphin (Fig. 4i). However, the decrease in DCFDA fluorescence of dorsomorphin-treated cells was lower in cultures incubated with serum compared to serum-starved cells (Fig. 4j, k). These data suggest that AMPK activity is required for ROS production in LX2 cells after *REST* KD, but other mechanisms induce ROS in serum starved LX2 cells following *REST* KD.

Next, we used this model to test if AMPK-dependent regulation of ROS is paralleled by changes in mitochondrial biogenesis. *REST* KD elevated mtDNA to genomic DNA ratio, and inhibition of AMPK failed to abrogate this effect (Fig. S3f). In contrast, serum starvation reversed the increase in mtDNA/genomic DNA ratio after *REST* KD, and AMPK inhibition exacerbated this effect (Fig. S3f). Together, these results suggest that serum starvation opposes *REST* KD-driven regulation of mitochondria biogenesis independent of AMPK activation.

AMPK is a key regulator of cellular energy homeostasis that can stimulate autophagy (Kim et al. 2011), a catabolic process that serves as an alternative energy source during nutrient deprivation. Accordingly, we asked whether autophagy in HSC is affected by *REST* KD and AMPK. Staining of phHSCs with monodansylcadaverine (MDC), a probe for acidic autophagic vacuoles, showed a significant increase in MDC fluorescence intensity following phHSCs *REST* KD (Fig. 4l), indicating that *REST* KD promoted autophagy. Further, *REST* KD promoted LC3a/b cleavage in LX2 cells (Fig. 4a, m), although no accumulation of MDC-stained autophagic vacuoles was observed in LX2 *REST* KD (Fig. S3f). AMPK was reported to promote autophagy by inducing ULK1 phosphorylation on Ser317^44^. We did not observe consistent increase in ULK1 Ser317 phosphorylation following *REST* KD in LX2 cells, suggesting that AMPK-driven activation of ULK1 is not a key pathway of autophagy induction following *REST* KD. Notably, the PI3K/mTORC1 signaling pathway, an essential regulator of HSC activation, has been shown to inhibit autophagy by promoting ULK1 phosphorylation at Ser757^44^. We observed a reduction in ULK1 Ser757 phosphorylation following *REST* KD in LX2 cells (Fig. 4a, m), suggesting that REST regulates autophagy through PI3K/mTORC1 pathway. Accordingly, we next investigated the role of PI3K/mTORC1 pathway in REST-dependent regulation of HSC activation.

### *REST* regulates PI3K/Akt/mTORC1 signaling cascade in activated HSCs

**Fig. 5:**
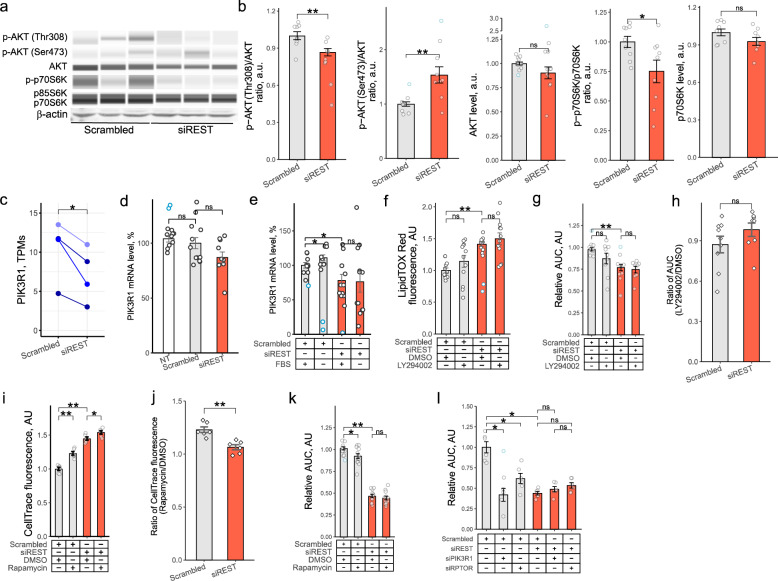
REST regulated growth of activated HSCs through PI3K/AKT/mTORC1 pathway.** a**,** b**,**d**,** e** LX2 cells were transfected with scrambled siRNA or siRNA targeting REST. 24 h later, cells were exposed to fresh medium containing DMEM with 10% FBS or 0% FBS for 72 h. **a**,** b** Western blotting of p-AKT (Ser473) (*n* = 9), p-AKT (Thr308) (*n* = 9), AKT (*n* = 12), p-p70S6K (*n* = 9) and p70S6K (*n* = 9) in LX2 cells. Representative images are shown. β-actin is shown as loading control. **(c)** RNA-sequencing. phHSCs were transfected with scrambled siRNA or siRNA targeting *REST* for 11 days and exposed to fresh DMEM with 10% FBS for 24 h. Dots show mRNA level of genes of interest in activated phHSCs normalized to *PPIA*, *RPLP0* and *SDHA* (*n* = 4). Colors indicate HSCs from individual donors (paired *t* test). **d**,** e** Bar graph of *PIK3R1* mRNA levels in LX2 cells. qPCR (*n* = 9–12). Bars show mean ± SEM, normalized to *PPIA*, *SDHA* and *RPLP0* and expressed relative to mean of scrambled siRNA-transfected cells (*t* test, Hommel test for multiple comparison correction). **f-l** LX2 cells were transfected with scrambled siRNA or siRNA targeting *REST* for 24 h and exposed to fresh DMEM with 10% FBS and DMSO (vehicle), LY294002 (10 µM) or rapamycin (5–10 nM) for 72 h. **f** Bar graph of LipidTOX Red staining of LX2s (*n* = 12). Samples were normalized to LX2 cells transfected with scrambled siRNA incubated in DMEM with 10% FBS (*t* test, Hommel test for multiple comparison correction). **g** Bar graph of relative LX2 growth (*n* = 9) presented in Fig. S4b. LX2 cells were incubated in IncuCyte for 72 h in DMEM with 10% FBS and photographed every 4 h. Y axis shows area under the curve (AUC) normalized to mean of scrambled siRNA exposed to vehicle. Bar show mean ± SEM in arbitrary units, AU (*t* test, Hommel test for multiple comparison correction). **h** Bar graph showing ratios of *REST* siRNA-transfected samples to mean of scrambled siRNA-transfected samples (*t* test, Hommel test for multiple comparisons correction). **i** Bar graph of LX2 proliferation measure by flow cytometry. LX2 cells were loaded with CellTrace prior to transfection (*n* = 6) (*t* test with Hommel test for multiple comparisons correction). **j** Bar graph showing ratios of REST siRNA-transfected samples to mean of scrambled siRNA-transfected samples (*t* test, Hommel test for multiple comparisons correction). **k** Bar graph of relative LX2 growth (*n* = 12) presented in Fig. S4c. LX2 cells were incubated in IncuCyte for 72 h in DMEM with 10% FBS and photographed every 4 h. Y axis shows area under the curve (AUC) normalized to mean of scrambled siRNA exposed to vehicle. Bar show mean ± SEM in arbitrary units, AU (*t* test, Hommel test for multiple comparison correction). **l** LX2 cells transfected with scrambled siRNA or/and siRNA targeting *REST*, *PIK3R1* or *RPTOR* for 24 h, incubated in IncuCyte for 72 h in DMEM with 10% FBS and photographed every 4 h (*n* = 6). Y axis shows area under the curve (AUC) normalized to mean of scrambled siRNA exposed to vehicle. Bar show mean ± SEM of relative growth expressed in arbitrary units, AU (Mann-Whitney *U* test, Hommel test for multiple comparisons correction). Outliers are indicated in blue. *p < 0.05, **p < 0.01

PIK3R1, a gene encoding the regulatory subunit of PI3K p85α, is essential for HSC activation (Wang et al. 2015), and REST has previously been linked with PI3K (Kreisler et al. 2010; Singh et al. 2013; Chen et al. 2017; Yin et al. 2015) and mTOR (Cho et al. 2015; Soga et al. 2021). Activation of the PI3K/AKT/mTORC1 cascade requires PI3K-dependent phosphorylation of AKT at both Thr307 and Ser473. We observed that Ser473 phosphorylation of AKT was increased following *REST* KD, while Thr307 phosphorylation was reduced, suggesting lower AKT activity following *REST* KD. To test whether activity of mTORC1 downstream of AKT was affected, we evaluated phosphorylation of p70S6K Thr389, a target of mTORC1. Similarly to mTORC1-dependent phosphorylation of ULK1 (Fig. 4a, m), phosphorylation of p70S6K Thr389 was reduced following *REST* KD (Fig. 5a, b), indicating that activity of the PI3K/AKT/mTORC1 cascade was downregulated. Further, *PIK3R1* mRNA levels were significantly reduced by *REST* KD in phHSCs (Fig. 5c) and in LX2 cells (Fig. 5d, e), indicating that REST regulated activity of the PI3K/AKT/mTORC1 cascade.

To study whether REST regulated HSC metabolism and growth through the PI3K/AKT/mTORC1 cascade, we exposed LX2 cells to the PI3K inhibitor LY294002 or mTORC1 inhibitor rapamycin. Inhibition of PI3K by LY294002 exposure did not significantly affect *REST* KD-driven lipid accumulation in LX2 cells (Fig. 5f), indicating that *REST*-mediated regulation of lipid metabolism in activated HSCs was independent of PI3K activity. LY294002 exposure did not significantly suppress LX2 growth following *REST* KD as measured in an IncuCyte^®^ live cell imaging system (Fig. 5g, h, Fig. S4b). Inhibition of mTORC1 using rapamycin attenuated cell division in LX2 cells exposed to either siRNA targeting *REST* or scrambled siRNA as measured by CellTrace fluorescence (Fig. 5i). Of note, the relative attenuation was significantly lower in *REST* KD cells (Fig. 5j), and rapamycin exposure did not affect LX2 confluence after *REST* KD (Fig. 5k, Fig. S4c), suggesting that *REST* KD reduced proliferation partially through mTORC1. Since LY294002 targets a multitude of PI3K subunits, we next asked whether subunit p85α, encoded by the gene PIK3R1 and previously implicated in control of activated HSC proliferation (Wang et al. 2015), was critical for the *REST* KD-driven effects. Knock down of either *PIK3R1* or *RAPTOR*, a key component of mTORC1 protein complex, reduced cell growth in scrambled siRNA-transfected cells (Fig. 5l, Fig. S4a). In contrast, knock down of either *PIK3R1* or *RAPTOR* failed to further attenuate LX2 growth following *REST* KD, indicating that REST controlled LX2 proliferation through the PIK3R1/Akt/mTORC1 cascade (Fig. 5l). Together, these observations indicate that REST controls proliferation of activated HSCs through the PI3K/AKT/mTORC1 cascade.

## Discussion

Here, we describe an essential role for REST in promoting proliferation of activated HSCs, a key process in the pathophysiology of metabolic dysfunction-associated liver diseases and liver regeneration.

It is well established that liver damage induces activation and proliferation of HSCs, and collagen synthesis and release is one of key functions of activated HSCs in the pathophysiology of liver fibrosis (Tsuchida and Friedman 2017; Liu et al. 2013). A plethora of extracellular signals promote various aspects of HSC activation through different mechanisms. For instance, TGFβ is a key pro-fibrogenic stimulus and growth factors such as PDGF are essential drivers of HSC proliferation (Tsuchida and Friedman 2017; Liu et al. 2013). The findings here indicate that REST functionally connects aspects of HSC activation as evidenced by the observations that *REST* KD reduced COLA1A expression and secretion, reduced proliferation, and promoted apoptosis. Further, serum starvation, but not TGFβ exposure upregulated *REST*, suggesting that HSC activation regulates REST function through both transcriptional and post-translational mechanisms. Paradoxically, although *REST* KD downregulated COL1A1 expression and secretion, intracellular levels of COL1A1 protein were elevated. This discrepancy could be explained by independent regulation of transcription, translation and protein secretion by REST. We speculate that reduced secretion of COL1A1 protein following *REST* KD could be the cause of accumulation of COL1A1 protein in intracellular depots. This accumulation may lead to ER stress and contribute to the elevated cell death we observed following *REST* KD. We observed that REST induced cell death through a caspase-dependent mechanism, and blocking caspase activity reduced *REST* KD-driven death. However, caspase inhibition did not affect HSC growth, indicating that REST-driven regulation of survival is not sufficient to explain reduction in growth after *REST* KD.

Although *REST* KD affected expression of HSC activation-associated genes *COL1A1* and *ACTA2*, expression of *PPARG* was unchanged. PPARγ promotes HSC quiescence, and activation of PPARγ reverted HSC activation (Hazra et al. 2004). Accordingly, our observations indicate that, although *REST* KD in activated HSCs reduces expression of activated HSC markers and reduces proliferation, it did not lead to restoration of the quiescent phenotype.

HSC activation involves dramatic changes in cell metabolism and these changes drive HSC activation (Tsuchida and Friedman 2017; Fondevila et al. 2022; Fondevila et al. 2024). ATP production through β-oxidation of intracellular lipid deposits and mitochondrial respiration is a critical energy source for activated HSCs (Fondevila et al. 2022). Interestingly, loss of *REST* induced accumulation of lipids in phHSCs and LX2 cells, indicating that utilization of lipids required for activation was affected. In line with this, we observed a reduction in the amount of V − ATP5A, higher proton leakage in mitochondria, reduced coupling efficiency, and accumulation of mitochondria with lower membrane potential. These observations suggest that REST is necessary for lipid utilization in activated HSCs and regulates mitochondrial metabolism necessary for sustaining HSC activation.

Interestingly, we also observed an increase in overall mitochondrial content of LX2 cells following *REST* KD. We speculate that this observation may indicate that accumulation of mitochondria with lower membrane potential after *REST* KD triggers a compensatory mechanism aimed at restoring mitochondria function. The mechanism would enable cells to partially restore continued cell proliferation following *REST* KD. AMPK was shown to promote mitochondrial health and biogenesis and, although we did not observe higher AMPK phosphorylation following *REST* KD, exposure of LX2 cells to the AMPK inhibitor dorsomorphin further reduced cell confluence and induced death after *REST* KD, indicating that AMPK activity was required to sustain cell growth and survival in cells without REST. However, inhibition of AMPK did not abrogate *REST* KD-driven increase in mitochondrial to genomic DNA ratio, suggesting that other mechanisms are responsible for this effect.

Exposure of activated HSCs to AMPK activators such as AICAR (Caligiuri et al. 2008) or serum starvation (Kain Ching et al. 2010) is known to arrest HSC growth, and our experiments confirmed this. However, *REST* KD did not abrogate growth of activated HSCs by activating AMPK-dependent signaling. Exposure of LX2 cells to an AMPK inhibitor after *REST* KD did not rescue LX2 growth, and activation of AMPK using AICAR was equally effective in LX2 cells with and without *REST* KD. Accordingly, other mechanisms reduce growth following *REST* KD.

Of note, these observations may explain the mechanistic difference between regulation of HSC growth by *REST* KD and serum starvation, as serum starvation-driven inhibition of HSCs proliferation is AMPK-dependent. Although we observed that both serum starvation and *REST* KD reduced growth of activated HSCs, these effects were additive in both phHSCs and LX2 cells. Further, serum starvation reduced mtDNA to genomic DNA ratio and restored ROS amounts after *REST* KD, suggesting that serum starvation inhibited the compensatory mechanism that was triggered in activated HSCs in response to *REST* KD. Together, these observations indicated that serum starvation and *REST* KD inhibit proliferation of activated HSCs through distinct mechanisms.

The PI3K/AKT/mTORC1 pathway is critical in activation of HSCs (Wang et al. 2015), and we observed that REST plays a key role in sustaining the activity of this cascade. *REST* KD reduced activity of the PI3K/AKT/mTORC1 pathway as evidenced by the reduced phosphorylation of its downstream targets ULK1 and p70S6K. Together with the marked increase in cleaved LC3a/b after *REST* KD, these observations suggest that *REST* KD promotes autophagy through downregulation of mTORC1-driven inhibition of ULK1 activity. In contrast, we did not observe higher AMPK-dependent phosphorylation of ULK1 following *REST* KD, indicating that autophagy in these cultures was not induced through AMPK-dependent activation of ULK1. Accordingly, the observed induction of autophagy following *REST* KD is at least partly driven by inhibition of the PI3K/AKT/mTORC1 pathway.

## Conclusions

In summary, our study uncovers REST as a previously unrecognized factor required for HSC activation and growth. REST KD in activated HSCs disrupts lipid utilization and mitochondrial metabolism, promotes autophagy and reduces growth, indicating that REST links activation-driven changes in activated HSC metabolism with growth. The findings identify REST in activated HSCs as a novel potential target of interest for further exploration the contexts of metabolic-associated liver diseases and liver regeneration.

## Supplementary Information

Supplementary Material 1.

## Data Availability

The data generated in this study (RNA-sequencing of phHSCs) has been deposited in the Gene Expression Omnibus (GEO) database with accession number GSE289433. The code is publicly available at [https://github.com/ImmunoBioLab/Shavva2025] (https:/github.com/ImmunoBioLab/Shavva2025).
