# Correction: Proliferation of activated hepatic stellate cells requires REST

**DOI:** 10.1186/s10020-026-01480-x

**Published:** 2026-05-11

**Authors:** Vladimir S. Shavva, L. Tarnawski, W. Dai, N. Moruzzi, A.-S. Haller, F. Borg, S. Hansson, Q. Guo, M. Cai, E. Fekete, J.J. Vacquié, A. Maestri, T. Liu, R.S. Vimaladithan, S.G. Malin, P. Saliba-Gustafsson, P.-O. Berggren, C.E. Hagberg, O. Ahmed, Peder S. Olofsson

**Affiliations:** 1 Department of Medicine, Laboratory of Immunobiology, Center for Bioelectronic Medicine, Karolinska Institutet, Solna, Stockholm Sweden; 2 Division of Cardiovascular Medicine, Department of Medicine, Center for Molecular Medicine, Karolinska Institutet, Karolinska University Hospital, Solna, Stockholm Sweden; 3 Department of Molecular Medicine and Surgery, The Rolf Luft Research Center for Diabetes and Endocrinology, Karolinska Institutet, Stockholm, Sweden; 4 Department of Chemistry and Applied Biosciences, Institute of Pharmaceutical Sciences, ETH Zurich, Zurich, Switzerland; 5 Cardio Metabolic Unit, Department of Laboratory Medicine, Department of Medicine, Karolinska Institutet, Huddinge, Stockholm Sweden; 6 Medicine Unit of Endocrinology, Theme Inflammation and Ageing, Karolinska University Hospital, Stockholm, Sweden; 7 Department of Biochemistry, College of Medicine and Medical Sciences, Arabian Gulf University, Manama, Kingdom of Bahrain; 8 Institutes of Bioelectronic Medicine, The Feinstein Institutes for Medical Research, Manhasset, NY USA

**Correction: Molecular Medicine 32, 28 (2026)**

**https://doi.org/10.1186/s10020-025-01406-z**

In this article (Shavva et al. 2026), the figures panel Figs. 1b and j and 2c appeared incorrectly and have now been corrected in the original publication. For completeness and transparency, both correct and incorrect versions are displayed below.

The original article has been corrected.

Incorrect Figure.

**Fig. 1:**
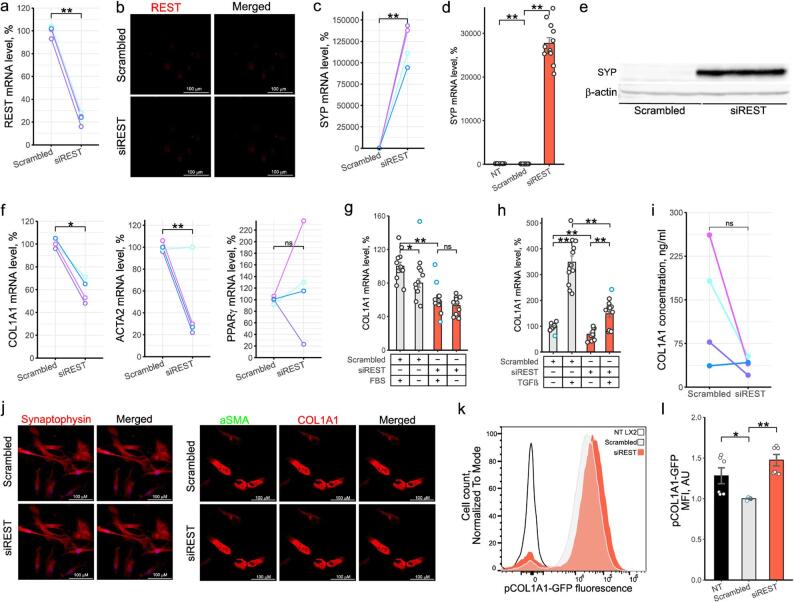
REST regulated growth of activated HSCs. **a**-**c**, f, i, j Primary human HSCs were activated by plating and transfected with scrambled siRNA or siRNA targeting REST for 12 days. a, c,f qPCR. Dots show mRNA level of genes of interest in activated phHSCs normalized to PPIA, RPLP0 and SDHA (*n* = 4). Colors indicate HSCs from individual donors (paired t test). **b** Confocal microscopy. Representative images of REST protein level in activated phHSC (*n* = 4). **d** qPCR. SYP expression in LX2 cells following 96 h transfection with scrambled siRNA or siRNA targeting REST (*n* = 12). Bars show mean ± SEM, normalized to PPIA, SDHA and RPLP0 and expressed relative to mean of scrambled siRNA-transfected cells (t test, Hommel test for multiple comparisons correction). **e** Western blotting. SYP protein level in LX2 cells following 96 h transfection with scrambled siRNA or siRNA targeting REST (*n* = 6). **g** qPCR. COL1A1 expression in LX2 cells following 96 h transfection with scrambled siRNA or siRNA targeting REST. Cells were exposed to media containing 10% or 0% FBS for 72 h (*n* = 11). Bars show mean ± SEM, normalized to PPIA, SDHA and RPLP0 and expressed relative to mean of scrambled siRNA-transfected cells (t test, Hommel test for multiple comparisons correction). **h** qPCR. COL1A1 expression in LX2 cells following 96 h transfection with scrambled siRNA or siRNA targeting REST. 24 h after transfection, cells were serum starved for 24 h and exposed to 5 ng/mL of TGFβ for 48 h (*n* = 12). Bars show mean ± SEM, normalized to PPIA, SDHA and RPLP0 and expressed relative to mean of scrambled siRNA-transfected cells (t test, Hommel test for multiple comparisons correction). **i** Quantification of COL1A1 protein secretion from activated phHSCs by ELISA (*n* = 4). Dots show REST protein level. Colors indicate HSCs from individual donors (paired t test). **j** Confocal microscopy. Representative images of COL1A1, αSMA and SYP protein level in phHSCs (*n* = 4). Merged – images showing nuclear staining (DAPI) and protein stainings. **k**-**l** Flow cytometry. LX2 carrying EGFP under control of COL1A1 promoter were transfected with scrambled siRNA or siRNA targeting REST for 96 h. NT LX2 – non-transfected cells. k Representative histogram of GFP fluorescence (*n* = 6). y axis shows cell count normalized to mode. l Bar graph showing GFP fluorescence (*n* = 6). The Y axis shows MFI (mean fluorescence intensity) normalized to scrambled siRNA-transfected cells (Mann-Whitney U test, Hommel test for multiple comparisons correction). NT – non-transfected. Outliers are indicated in blue. *p < 0.05, **p < 0.01

**Fig. 2:**
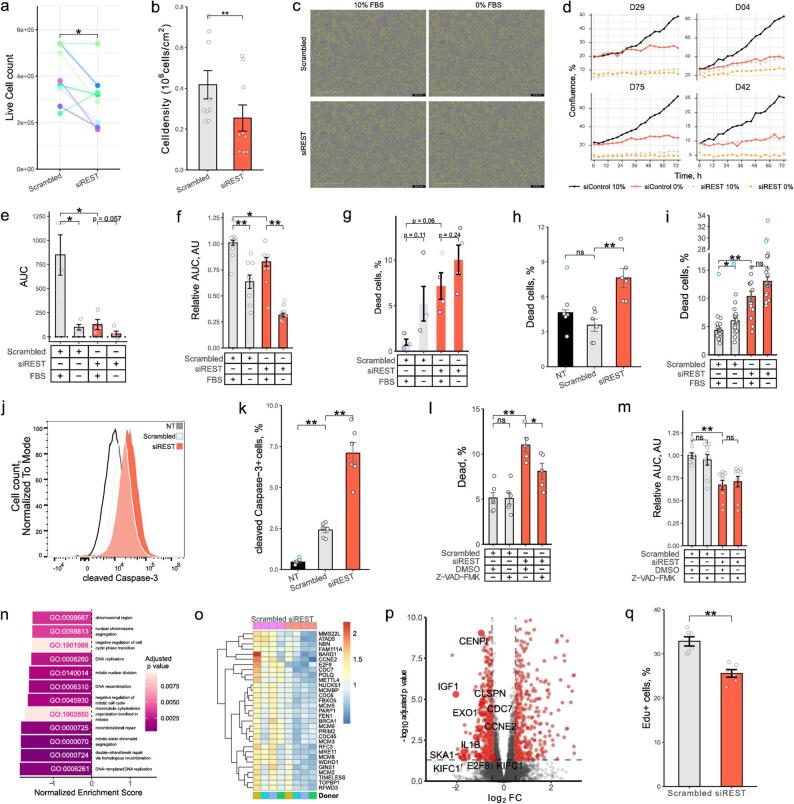
REST regulates survival and proliferation of HSCs. **a** Primary human HSCs were activated by plating and transfected with scrambled siRNA or siRNA targeting REST for 6 days (*n* = 8). Live cell counts were quantified using trypan blue staining (paired t test). **b** LX2 cells were transfected with scrambled siRNA or siRNA targeting REST for 96 h (*n* = 9). Live cell counts were quantified using trypan blue staining (t test). **c**-**e** Analysis of phHSC growth using IncuCyte (*n* = 4). PhHSCs were transfected with scrambled siRNA or siRNA targeting REST. After 6 days cells were passaged, re-transfected and incubated with 10% FBS for 48 h. Next, cells were placed into IncuCyte and media was changed to DMEM or DMEM + 10% FBS and photographed for 72 h every 4 h. **c** Representative images of primary human HSCs from D29 following 12 d of activation and siRNA transfection. Yellow shows cell borders. **d** Change in confluence. Y axis shows confluence (%), and x axis shows time, donor ID indicated on top. **e** Area under the curve (AUC). Confluence was normalized to confluence at day 0 per well. Bars show mean AUC ± SEM (Mann-Whitney U test, FDR multiple comparison correction test). **f**, m Bar graph of relative LX2 growth (f: *n* = 9, m: *n* = 9) presented in Fig. S2b. LX2s were transfected with siRNA for 24 h and incubated in IncuCyte for 72 h. Photographs were taken every 4 h. Y axis shows area under the curve (AUC) normalized to mean of scrambled siRNA exposed to 10% FBS (Mann-Whitney U test, Hommel test for multiple comparison correction). **g** Bar graph of phHSC viability following activation and REST KD (*n* = 4). Dead cells were quantified using zombie yellow staining and flow cytometry. Bars show mean ± SEM % of dead cells from single cell gate, dots indicate individual donors (paired t test with FDR test for multiple comparisons correction). **h**, **i**, l Bar graph of LX2 viability after REST KD (h, l: *n* = 6, i: *n* = 18). Dead cells were quantified by propidium iodide (i) or zombie yellow (h, i, l) staining and flow cytometry. Bars show mean ± SEM % of dead cells from single cell gate (Mann-Whitney U test, Hommel test for multiple comparisons correction). **j**, **k** Flow cytometry. Representative histogram (j) and bar graph (k) of cleaved Caspase-3 fluorescence (*n* = 6). Bars show mean ± SEM % of cells from single cell gate (Mann-Whitney U test, Hommel test for multiple comparisons correction). **l**, **m** LX2 cells were transfected with scrambled siRNA or siRNA targeting REST for 24 h and exposed to DMSO (vehicle) or Z-VAD-FMK (10 µM) for 72 h. **n**-**o** RNA-sequencing analysis of phHSC transcriptomes following 12 d activation and transfection (*n* = 4). Cells were exposed to fresh DMEM with 10% FBS for 24 h before harvesting. **n** Bar graph of normalized enrichment scores (NES) of GO terms related to proliferation. Gene Set Enrichment Analysis (GSEA) was performed with p value cutoff of 0.05. Color indicates adjusted p value. **o** Heatmap of transcripts per million (TPM) for genes belonging to GO:0006261 “DNA-templated DNA replication”. TPMs were normalized to average of TPMs per gene. **p** Volcano plot of top 10 most affected genes belonging to proliferation pathways shown in Fig. 2n. Horizontal dashed line indicates p value cutoff of 0.05, vertical dashed line indicates log2 fold change cutoff of 0.5. **q** Bar graph of LX2 proliferation measure by EdU staining and flow cytometry (*n* = 6) (t test with Hommel test for multiple comparisons correction). NT – non-transfected. Outliers are indicated in blue. *p < 0.05, **p < 0.01

Correct Figure.

**Fig. 1:**
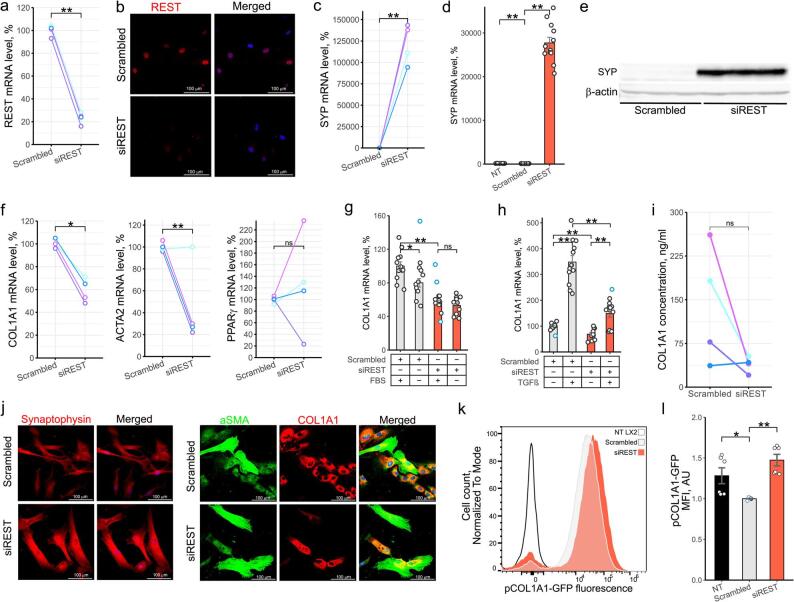
REST regulated growth of activated HSCs. **a**-**c**, f, i, j Primary human HSCs were activated by plating and transfected with scrambled siRNA or siRNA targeting REST for 12 days. a, c,f qPCR. Dots show mRNA level of genes of interest in activated phHSCs normalized to PPIA, RPLP0 and SDHA (*n* = 4). Colors indicate HSCs from individual donors (paired t test). **b** Confocal microscopy. Representative images of REST protein level in activated phHSC (*n* = 4). **d** qPCR. SYP expression in LX2 cells following 96 h transfection with scrambled siRNA or siRNA targeting REST (*n* = 12). Bars show mean ± SEM, normalized to PPIA, SDHA and RPLP0 and expressed relative to mean of scrambled siRNA-transfected cells (t test, Hommel test for multiple comparisons correction). **e** Western blotting. SYP protein level in LX2 cells following 96 h transfection with scrambled siRNA or siRNA targeting REST (*n* = 6). **g** qPCR. COL1A1 expression in LX2 cells following 96 h transfection with scrambled siRNA or siRNA targeting REST. Cells were exposed to media containing 10% or 0% FBS for 72 h (*n* = 11). Bars show mean ± SEM, normalized to PPIA, SDHA and RPLP0 and expressed relative to mean of scrambled siRNA-transfected cells (t test, Hommel test for multiple comparisons correction). **h** qPCR. COL1A1 expression in LX2 cells following 96 h transfection with scrambled siRNA or siRNA targeting REST. 24 h after transfection, cells were serum starved for 24 h and exposed to 5 ng/mL of TGFβ for 48 h (*n* = 12). Bars show mean ± SEM, normalized to PPIA, SDHA and RPLP0 and expressed relative to mean of scrambled siRNA-transfected cells (t test, Hommel test for multiple comparisons correction). **i** Quantification of COL1A1 protein secretion from activated phHSCs by ELISA (*n* = 4). Dots show REST protein level. Colors indicate HSCs from individual donors (paired t test). **j** Confocal microscopy. Representative images of COL1A1, αSMA and SYP protein level in phHSCs (*n* = 4). Merged – images showing nuclear staining (DAPI) and protein stainings. **k**-**l** Flow cytometry. LX2 carrying EGFP under control of COL1A1 promoter were transfected with scrambled siRNA or siRNA targeting REST for 96 h. NT LX2 – non-transfected cells. **k** Representative histogram of GFP fluorescence (*n* = 6). y axis shows cell count normalized to mode. **l** Bar graph showing GFP fluorescence (*n* = 6). The Y axis shows MFI (mean fluorescence intensity) normalized to scrambled siRNA-transfected cells (Mann-Whitney U test, Hommel test for multiple comparisons correction). NT – non-transfected. Outliers are indicated in blue. *p < 0.05, **p < 0.01

**Fig. 2:**
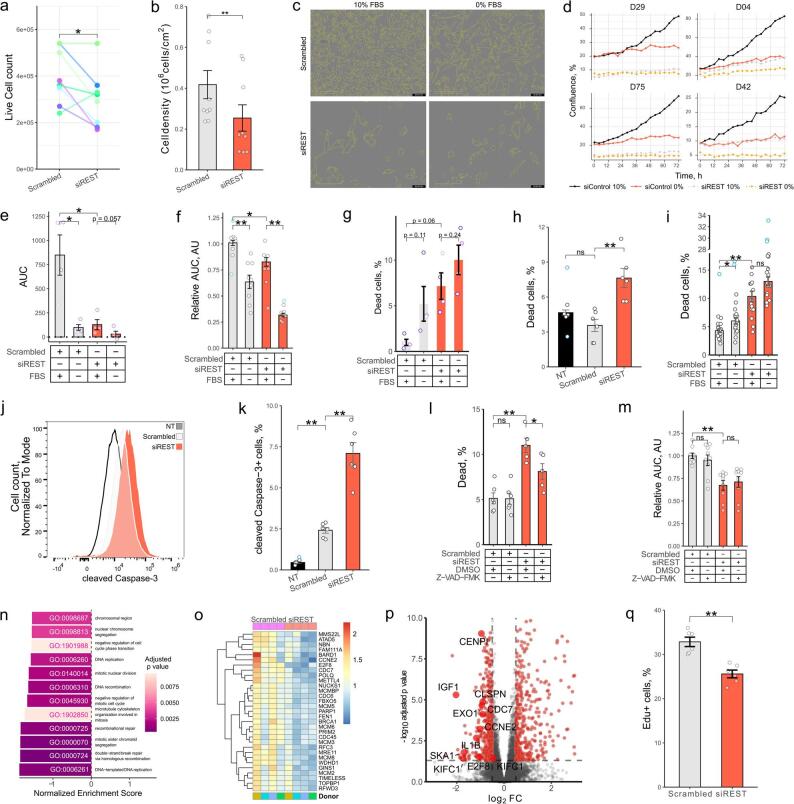
REST regulates survival and proliferation of HSCs. **a** Primary human HSCs were activated by plating and transfected with scrambled siRNA or siRNA targeting REST for 6 days (*n* = 8). Live cell counts were quantified using trypan blue staining (paired t test). **b** LX2 cells were transfected with scrambled siRNA or siRNA targeting REST for 96 h (*n* = 9). Live cell counts were quantified using trypan blue staining (t test). c-e Analysis of phHSC growth using IncuCyte (*n* = 4). PhHSCs were transfected with scrambled siRNA or siRNA targeting REST. After 6 days cells were passaged, re-transfected and incubated with 10% FBS for 48 h. Next, cells were placed into IncuCyte and media was changed to DMEM or DMEM + 10% FBS and photographed for 72 h every 4 h. **c** Representative images of primary human HSCs from D29 following 12 d of activation and siRNA transfection. Yellow shows cell borders. **d** Change in confluence. Y axis shows confluence (%), and x axis shows time, donor ID indicated on top. **e** Area under the curve (AUC). Confluence was normalized to confluence at day 0 per well. Bars show mean AUC ± SEM (Mann-Whitney U test, FDR multiple comparison correction test). **f**, m Bar graph of relative LX2 growth (f: *n* = 9, m: *n* = 9) presented in Fig. S2b. LX2s were transfected with siRNA for 24 h and incubated in IncuCyte for 72 h. Photographs were taken every 4 h. Y axis shows area under the curve (AUC) normalized to mean of scrambled siRNA exposed to 10% FBS (Mann-Whitney U test, Hommel test for multiple comparison correction). **g** Bar graph of phHSC viability following activation and REST KD (*n* = 4). Dead cells were quantified using zombie yellow staining and flow cytometry. Bars show mean ± SEM % of dead cells from single cell gate, dots indicate individual donors (paired t test with FDR test for multiple comparisons correction). **h**, **i**, l Bar graph of LX2 viability after REST KD (h, l: *n* = 6, i: *n* = 18). Dead cells were quantified by propidium iodide (i) or zombie yellow (h, i, l) staining and flow cytometry. Bars show mean ± SEM % of dead cells from single cell gate (Mann-Whitney U test, Hommel test for multiple comparisons correction). **j**, **k** Flow cytometry. Representative histogram (j) and bar graph (k) of cleaved Caspase-3 fluorescence (*n* = 6). Bars show mean ± SEM % of cells from single cell gate (Mann-Whitney U test, Hommel test for multiple comparisons correction). **l**, **m** LX2 cells were transfected with scrambled siRNA or siRNA targeting REST for 24 h and exposed to DMSO (vehicle) or Z-VAD-FMK (10 µM) for 72 h. **n**-**o** RNA-sequencing analysis of phHSC transcriptomes following 12 d activation and transfection (*n* = 4). Cells were exposed to fresh DMEM with 10% FBS for 24 h before harvesting. n Bar graph of normalized enrichment scores (NES) of GO terms related to proliferation. Gene Set Enrichment Analysis (GSEA) was performed with p value cutoff of 0.05. Color indicates adjusted p value. **o** Heatmap of transcripts per million (TPM) for genes belonging to GO:0006261 “DNA-templated DNA replication”. TPMs were normalized to average of TPMs per gene. **p** Volcano plot of top 10 most affected genes belonging to proliferation pathways shown in Fig. 2n. Horizontal dashed line indicates p value cutoff of 0.05, vertical dashed line indicates log2 fold change cutoff of 0.5. **q** Bar graph of LX2 proliferation measure by EdU staining and flow cytometry (*n* = 6) (t test with Hommel test for multiple comparisons correction). NT – non-transfected. Outliers are indicated in blue. *p < 0.05, **p < 0.01

## References

[CR1] Shavva VS, Tarnawski L, Dai W, et al. Proliferation of activated hepatic stellate cells requires REST. Mol Med. 2026;32:28. 10.1186/s10020-025-01406-z. 41606712 10.1186/s10020-025-01406-z PMC12924475

