# Supplementary material for: Proliferation of activated hepatic stellate cells requires REST
Source: Mol Med. 2026 Jan 28;32:28. doi: 10.1186/s10020-025-01406-z (PMC12924475; doi:10.1186/s10020-025-01406-z)
Supplement: Supplementary file 1 — Supplementary Material 1. [file 10020_2025_1406_MOESM1_ESM.docx]

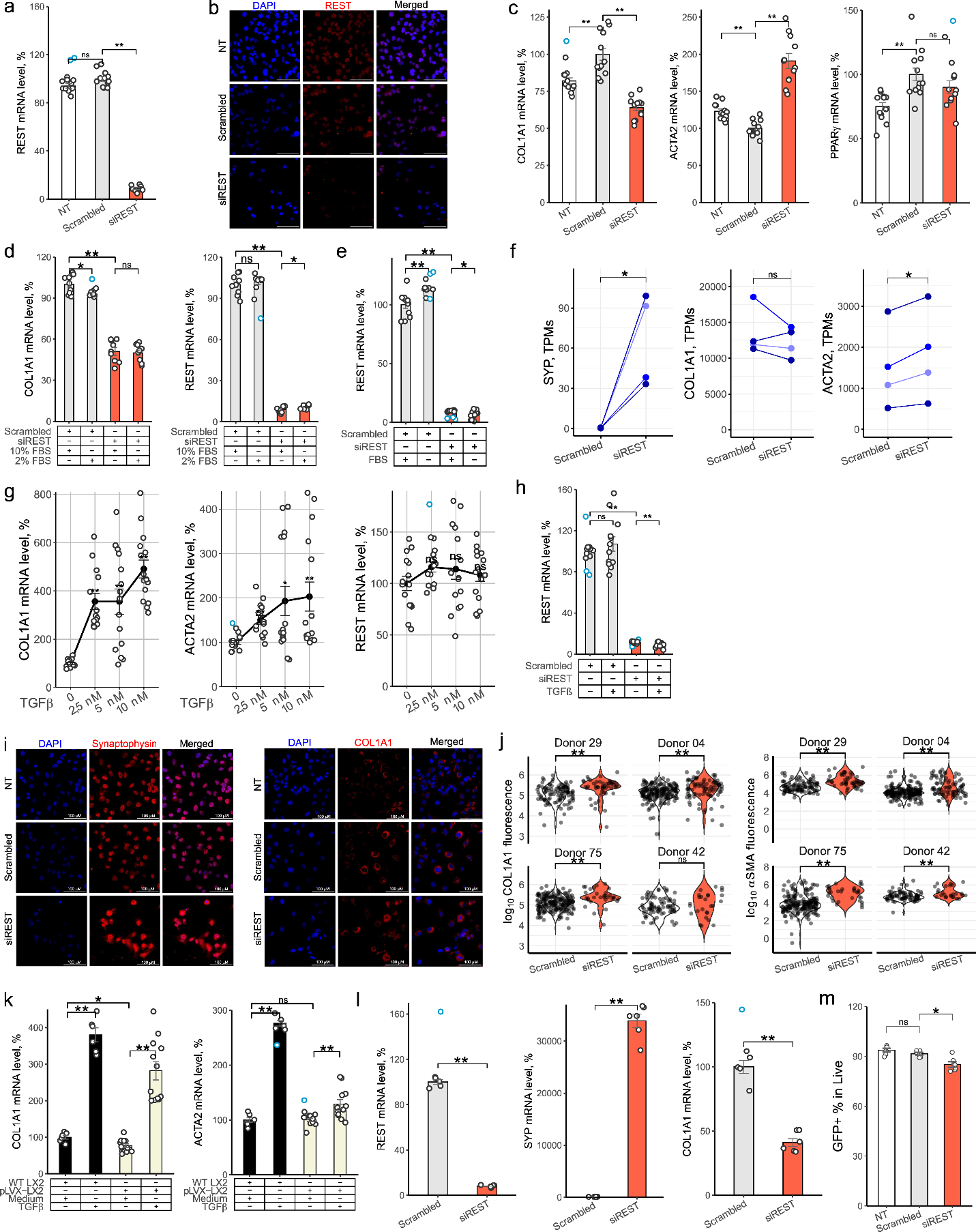


**Figure S1: REST regulates activation of LX2 cells. (a-e,g-i)** LX2 cells were transfected with scrambled or REST siRNA for 96h. **(a, c)** Gene expression following REST KD in LX2 cells (n=12). Bars show mean±SEM, normalized to *PPIA*, *SDHA* and *RPLP0* and expressed relative to mean of scrambled siRNA-transfected cells (*t* test, Hommel test for multiple comparison correction). **(b, i)** Representative images of REST (b), SYP (e) and COL1A1 (e) protein level in LX2 cells following siRNA transfection (n=12). Confocal microscopy. **(d, e)** qPCR. Gene expression in LX2 cells following 96h transfection with scrambled siRNA or siRNA targeting REST. Cells were exposed to media containing 10%, 2% or 0% FBS for 72h (n=9-11). Bars show mean±SEM, normalized to *PPIA*, *SDHA* and *RPLP0* and expressed relative to mean of scrambled siRNA-transfected cells (*t* test, Hommel test for multiple comparisons correction). **(f)** RNA-sequencing. mRNA levels of genes of interest in activated phHSCs (n=4). Cells were exposed to fresh DMEM with 10% FBS for 24h. y axis shows transcripts per million (TPM). Colors indicate HSCs from individual donors (t test). **(g)** Gene expression following in LX2 cells following TGFβ exposure. Cells were serum-starved for 24h and exposed to TGFβ for 48h (n=12). The Y axis shows TGFβ concentration. Bars show mean±SEM, normalized to *PPIA*, *SDHA* and *RPLP0* and expressed relative to mean of scrambled siRNA-transfected cells (*t* test, Hommel test for multiple comparison correction). **(h)** qPCR. *REST* expression in LX2 cells following 96h transfection with scrambled siRNA or siRNA targeting REST. 24h after transfection, cells were serum starved for 24h and exposed to 5 ng/mL of TGFβ for 48h (n=12). Bars show mean±SEM, normalized to *PPIA*, *SDHA* and *RPLP0* and expressed relative to mean of scrambled siRNA-transfected cells (*t* test, Hommel test for multiple comparisons correction). **(j)** Violin plots showing COL1A1 (g) and αSMA (h) protein level in activated phHSCs. Confocal microscopy (Mann-Whitney *U* test). **(k)** Bar graph of gene expression in LX2 and pLVX-LX2 cells following 48h exposure to 5 ng/mL of TGFβ (n=12-24). Bars show mean±SEM, normalized to PPIA, SDHA and RPLP0 and expressed relative to mean of LX2 cells (Mann-Whitney *U* test, Hommel test for multiple comparisons correction). **(l)** Bar graph of gene expression in pLVX-LX2 cells following transfection with scrambled or REST siRNA for 96h (n=6). Bars show mean±SEM, normalized to PPIA, SDHA and RPLP0 and expressed relative to mean of LX2 cells (Mann-Whitney *U* test, Hommel test for multiple comparisons correction). **(m)** Bar graph showing percentage of GFP^+^ LX2 cells following REST knock down (n=6). LX2 cells expressing EGFP under control of COL1A1 promoter were transfected with scrambled siRNA or siRNA targeting REST for 96h and GFP fluorescence was measured using flow cytometry (*t* test, Hommel test for multiple comparison correction). Outliers are indicated in blue. *p<0.05, **p<0.01.


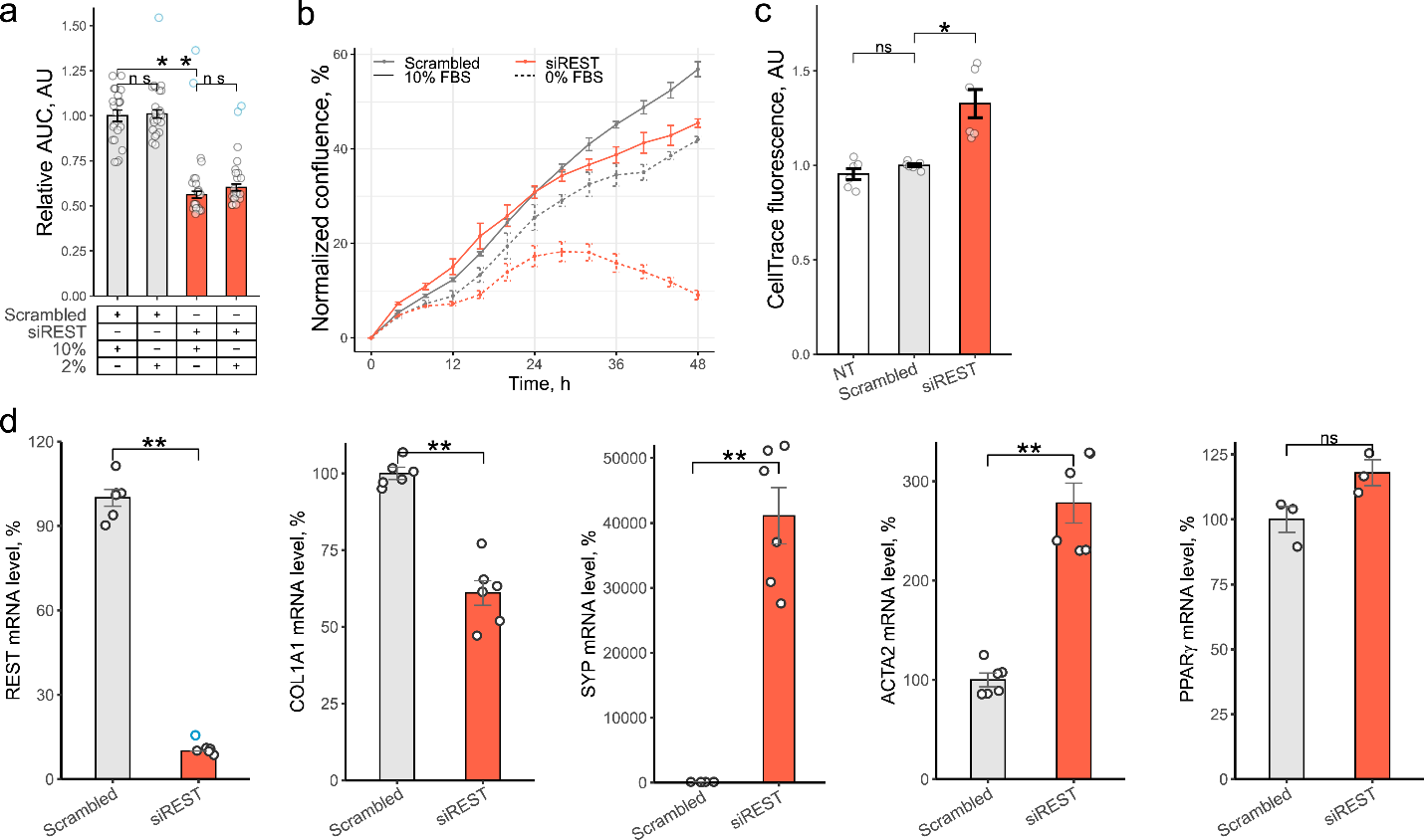


**Figure S2: REST regulates proliferation of LX2 cells.** LX2 cells were transfected with scrambled or REST siRNA and incubated in DMEM with 10% FBS for 96h. **(a)** Representative plot of change in LX2 confluence following *REST* KD. LX2 cells were transfected with scrambled siRNA or siRNA targeting REST and incubated in IncuCyte for 72h. in DMEM with 10% or 0% FBS. x axis shows time in hours, y axis shows normalized confluence in % of total area. Confluence was normalized to 0h per sample. **(b)** Bar graph of LX2 proliferation (n=6) measure by flow cytometry. LX2 cells were loaded with CellTrace, transfected with scrambled siRNA or siRNA targeting REST for 96h. CellTrace accumulation was measured using flow cytometry (t test, Hommel test for multiple comparisons correction). **(c)** Bar graph of relative LX2 growth (n=23). LX2s were transfected with siRNA for 24h and incubated in Incucyte for 72h. Photographs were taken every 4h. Y axis shows area under the curve (AUC) normalized to mean of scrambled siRNA exposed to 10% FBS (Mann-Whitney U test, Hommel test for multiple comparison correction). **(d)** Bar graph of gene expression in LX2 cells following transfection with scrambled or REST siRNA for 96h (n=6). Cells were trypsinized 24h before harvest and reseeded in new wells. Bars show mean±SEM, normalized to PPIA, SDHA and RPLP0 and expressed relative to mean of LX2 cells (Mann-Whitney *U* test, Hommel test for multiple comparisons correction). Outliers are indicated in blue. *p<0.05, **p<0.01.

**
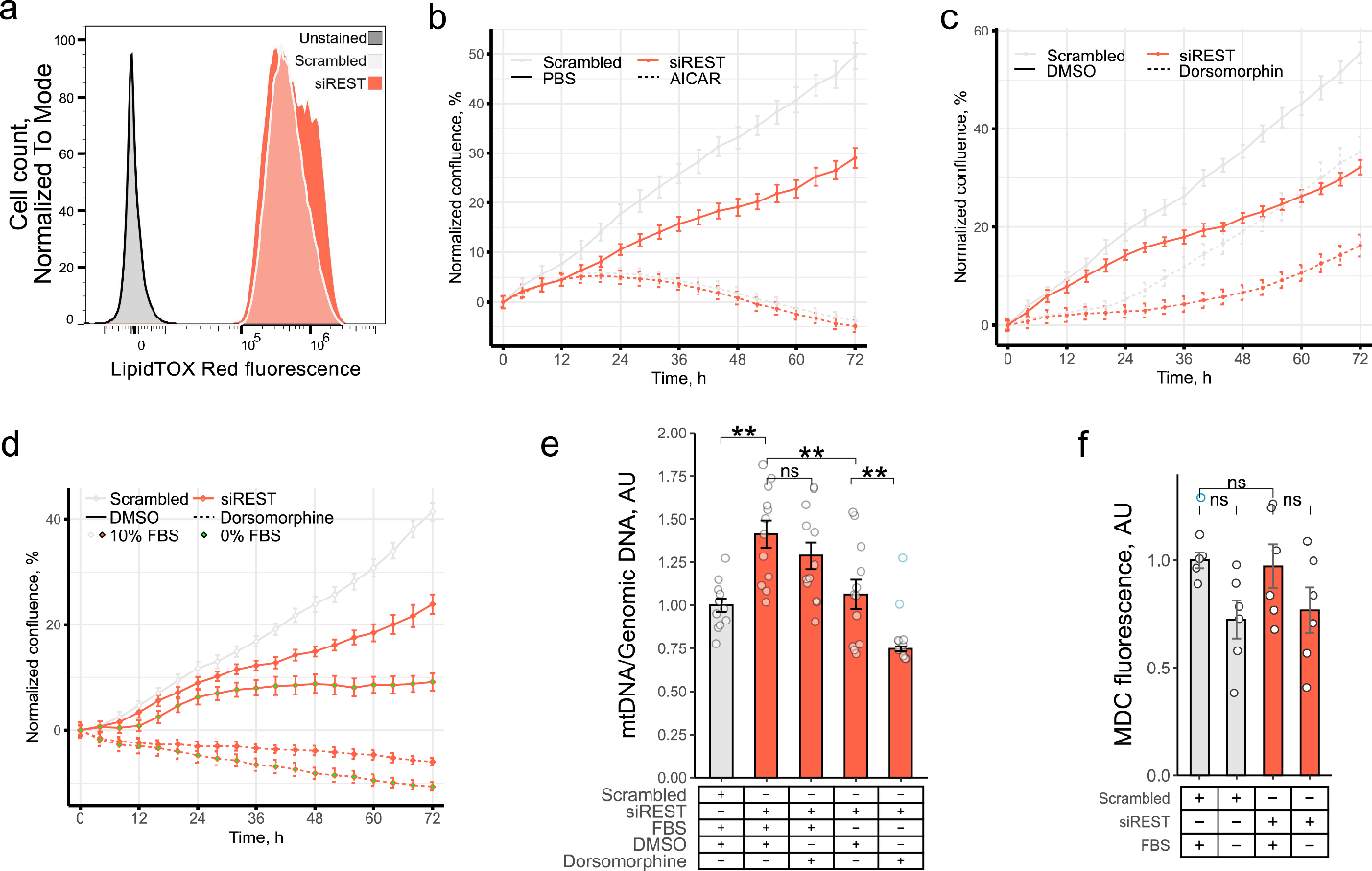
**

**Figure S3: REST links metabolism and growth in activated HSCs. (a)** Histogram of LipidTOX Red fluorescence following REST KD in phHSCs. PhHSCs were activated and transfected with scrambled siRNA or siRNA targeting REST for 12 days. Histogram shows concatenated samples of 4 individual donors per condition. X axis shows LipidTOX Red fluorescence, y axis showed cell count normalized to mode. Flow cytometry. **(b,c,e)** Representative plot of change in LX2 confluence following *REST* KD. LX2 cells were transfected with scrambled siRNA or siRNA targeting REST for 24h, exposed to DMSO (vehicle), AICAR (2 mM) or dorsomorphin (10 μM) and incubated in IncuCyte for 72h. in DMEM with or without 10% FBS. x axis shows time in hours, y axis shows normalized confluence in % of total area. Confluence was normalized to 0h per sample. **(d)** Bar graph of monodansylcadaverine (MDC) fluorescence in LX2 cells. LX2 cells were transfected with scrambled siRNA or siRNA targeting REST and incubated in DMEM with 10% or 0% FBS for 72h. y axis shows MDC fluorescence normalized to scrambled siRNA-transfected cells exposed to DMEM with 10% FBS (Mann-Whitney *U* test, Hommel test for multiple comparison correction). **(f)** Bar graphs of mitochondrial to genomic DNA ratio in LX2 cells (n=12). PCR. Samples were normalized to LX2 cells transfected with scrambled siRNA incubated in DMEM with 10% FBS (Mann-Whitney *U* test, Hommel test for multiple comparison correction). Outliers are indicated in blue. *p<0.05, **p<0.01.


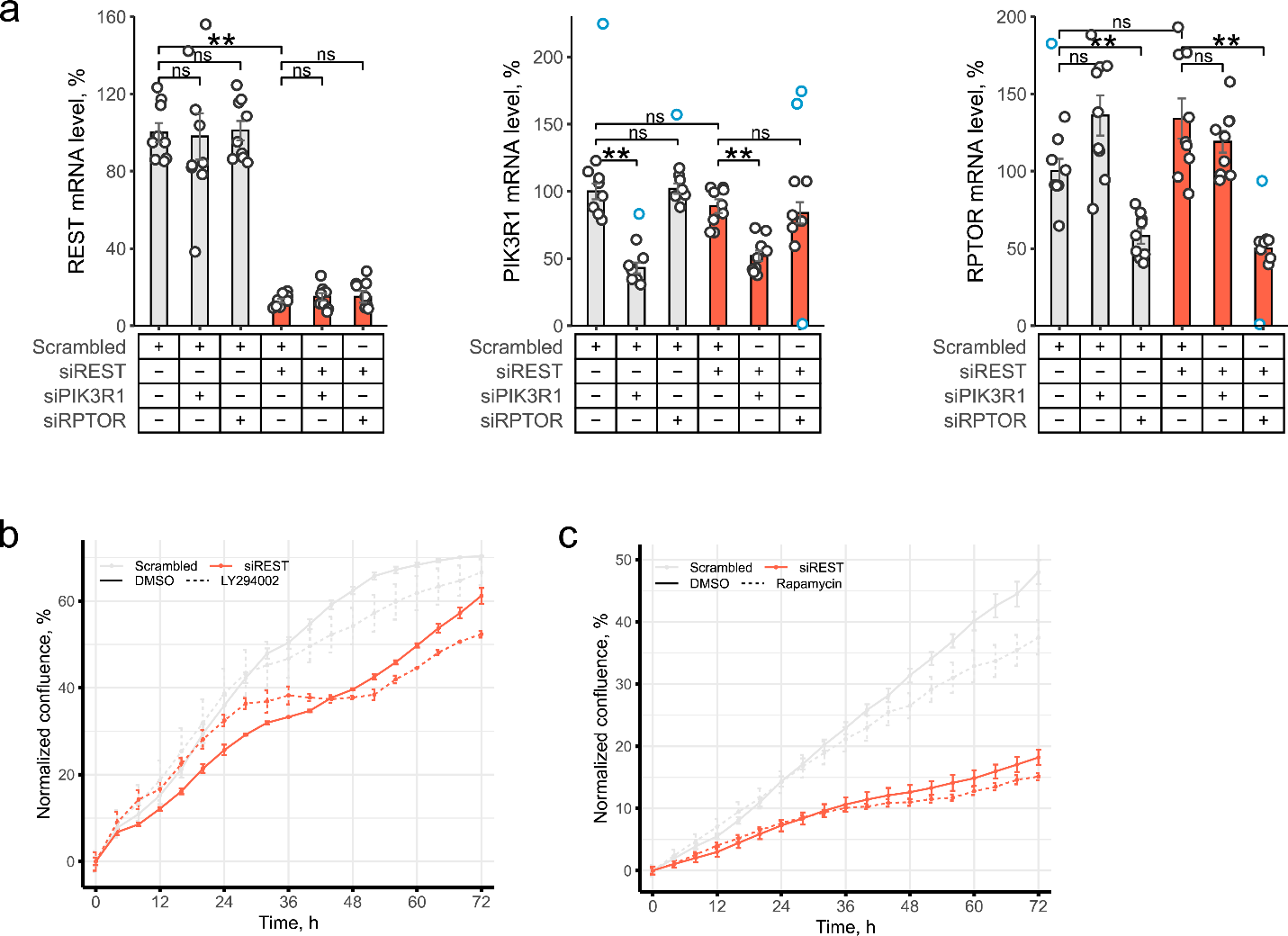


**Figure S4: REST regulates LX2 growth through PI3K/mTORC1 pathway. (a)** qPCR. LX2 cells were co-transfected with the combination of scrambled siRNA, siRNA targeting REST, PIK3R1 or RPTOR. 24h following transfection, cells incubated in DMEM with 10% FBS for 72h (n=9). Bars show mean±SEM, normalized to *PPIA*, *SDHA* and *RPLP0* and expressed relative to mean of scrambled siRNA-transfected cells (*t* test, Hommel test for multiple comparison correction ). **(b,c)** Representative plot of change in LX2 confluence following *REST* KD. LX2 cells were transfected with scrambled siRNA or siRNA targeting REST for 24h, exposed to DMSO (vehicle), LY294002 (10 μM) or rapamycin (5-10 nM) and incubated in IncuCyte for 72h. in DMEM with 10%. x axis shows time in hours, y axis shows normalized confluence in % of total area. Confluence was normalized to 0h per sample. Outliers are indicated in blue. *p<0.05, **p<0.01.

**
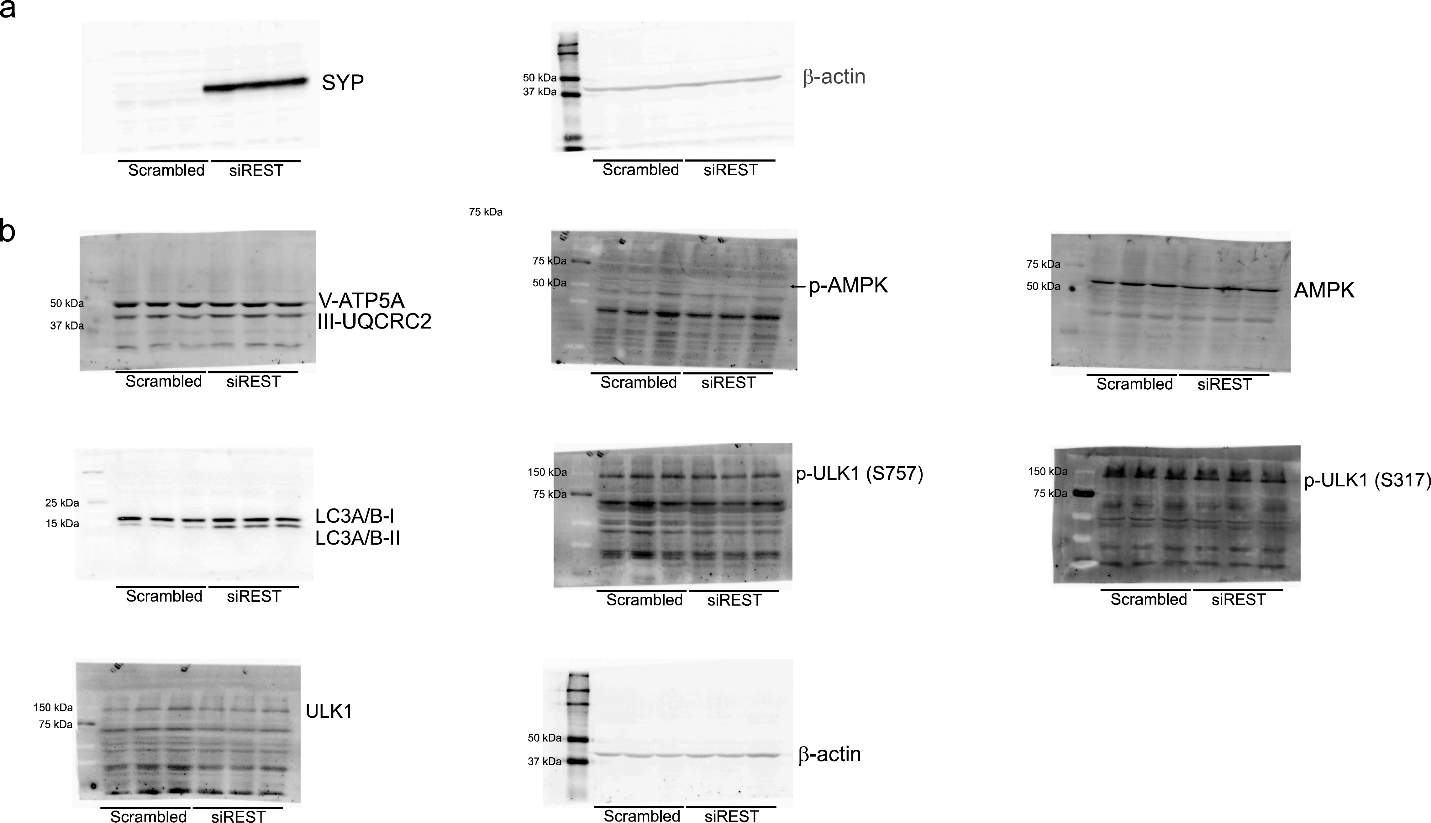
Figure S5: Original western blot images. (a)** For Fig. 1e. **(b)** For Fig. 4a.


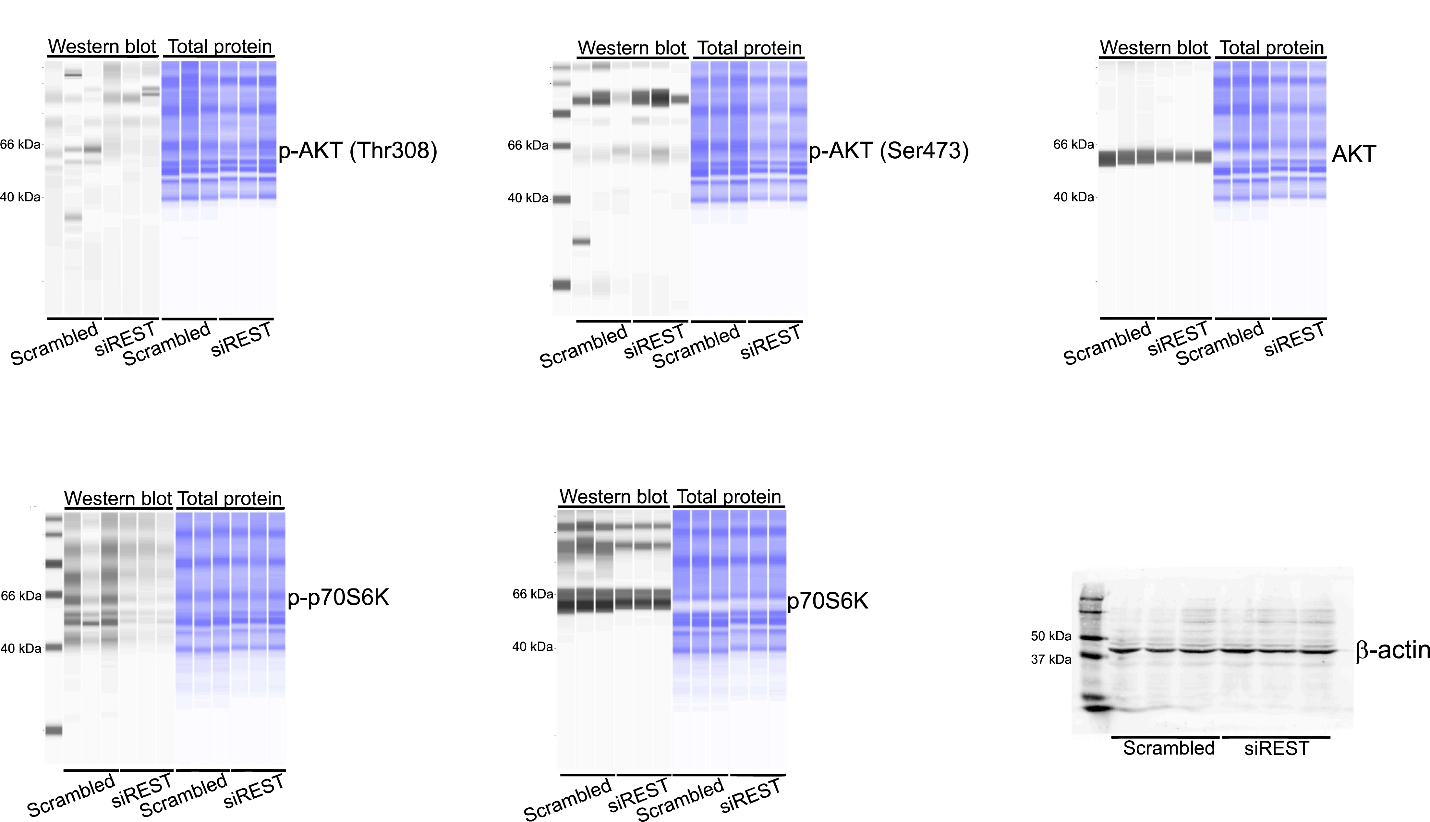


**Figure S6: Original western blot images for Fig. 5a.**


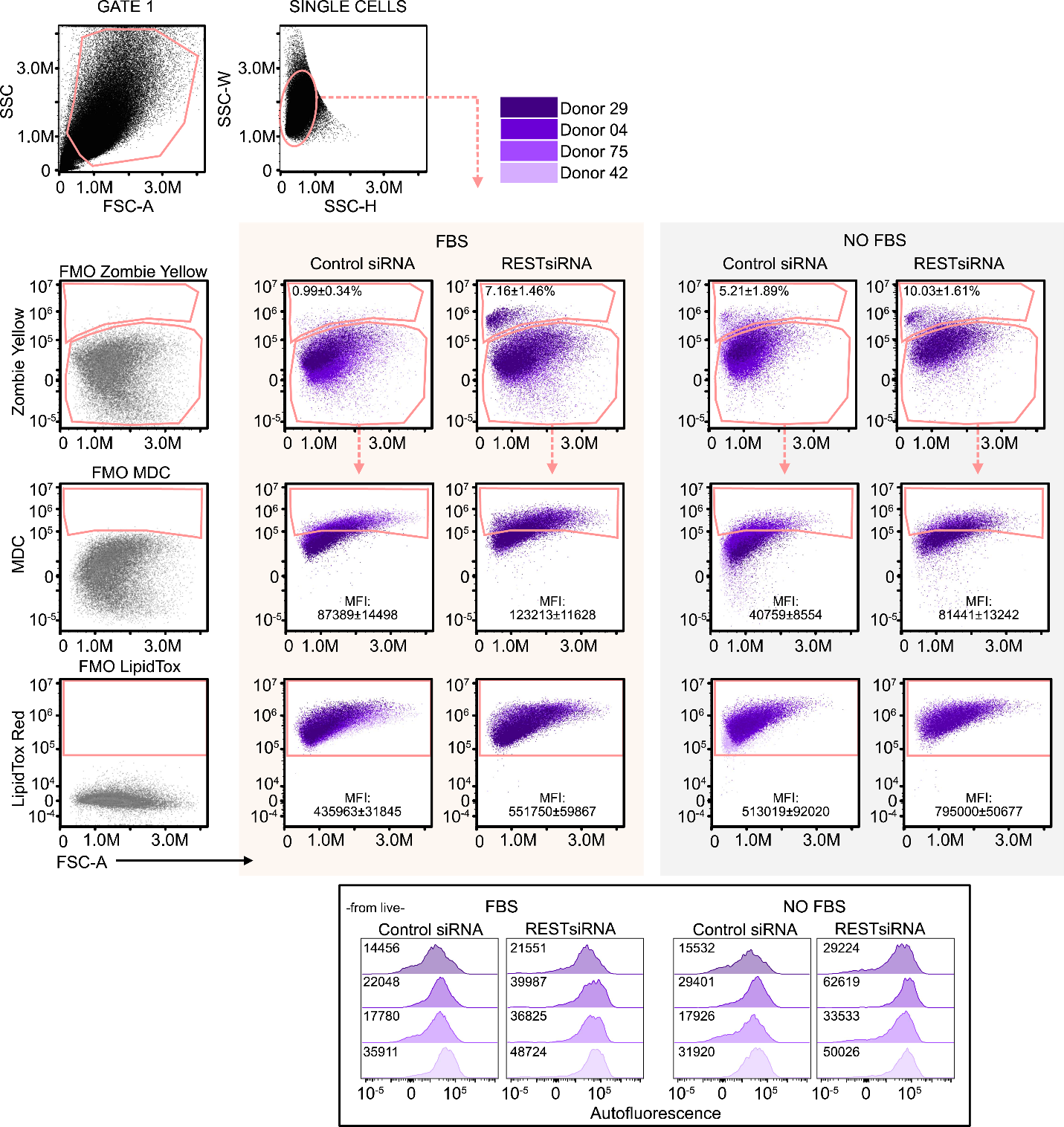


**Figure S7: Representative plots of flow cytometry gating and analysis of primary human HSCs** (n = 4). Primary human HSCs were activated by plating and transfected with scrambled siRNA or siRNA targeting REST for 12 days. Cells were stained with viability dye zombie yellow, dye for neutral lipids (LipidTOX Red) and monodansylcadaverine (MDC), a marker of autophagic vacuoles.


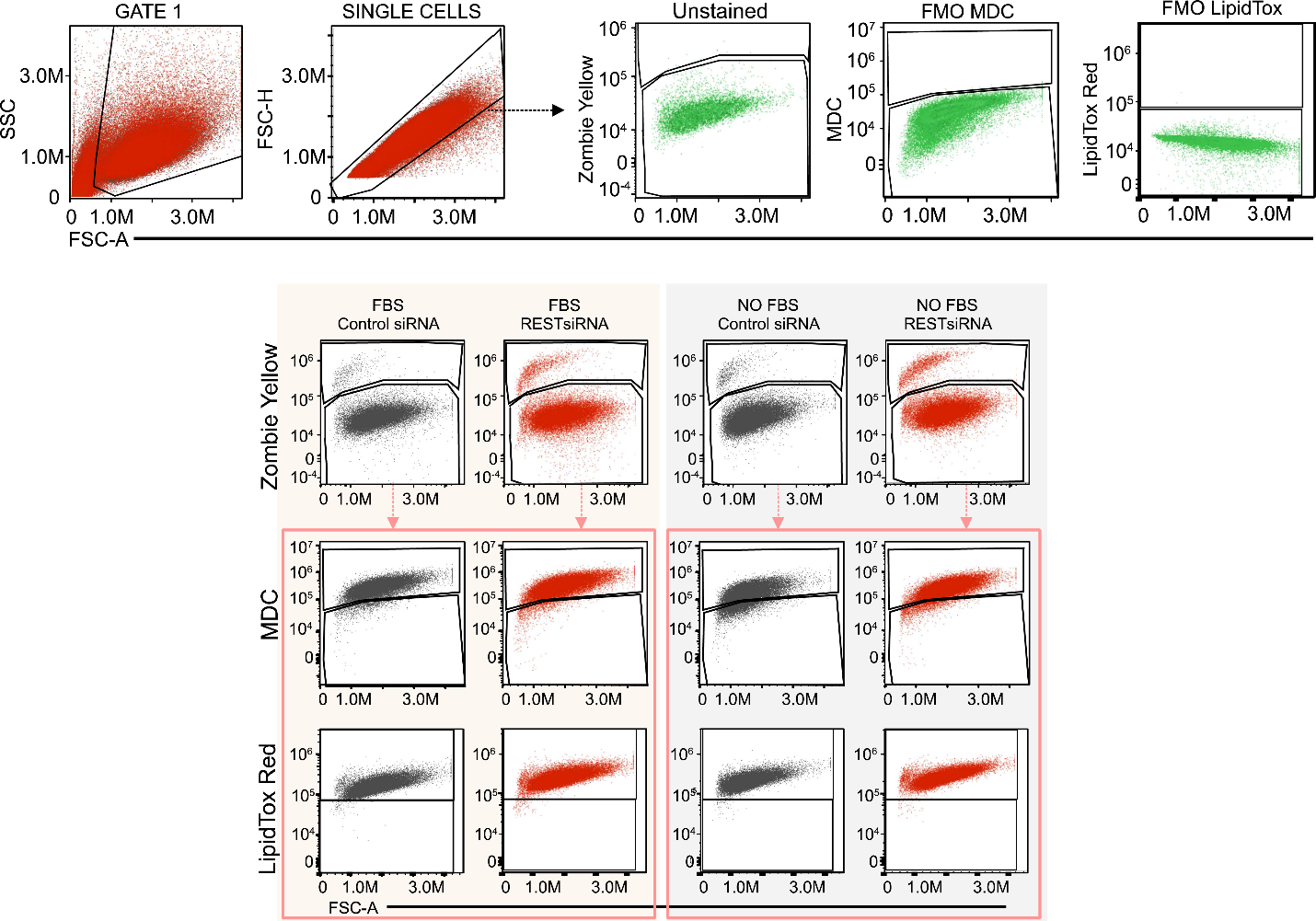


**Figure S8:** **Representative plots of flow cytometry gating and analysis of LX2 cells**. LX2 cells were transfected with scrambled siRNA or siRNA targeting REST and incubated in DMEM with 10% FBS or DMEM for 72h. Gating strategy following staining with viability dye zombie yellow, dye for neutral lipids (LipidTOX Red) and monodansylcadaverine (MDC), a marker of autophagic vacuoles.

## Supplementary Table 1: Primers

| **GOI** | **Forward (5'-3')** | **Reverse (5'-3')** | **Taqman probe (5'-3')** |
| --- | --- | --- | --- |
| RPLP0 | GACCTCCTTTTTCCAGGCTTTA | TTGATCAGCTGCACATCACTC | TCCAGGGGCACCATTGAAATCC |
| SDHA | CAAGGCGAAAGGTTTATGGA | CAGGGCCACAGCCTCTT | CCCTGTCGCGAAGGACCTGG |
| PIK3R1 | GCAATGAGAAAGAAATACAAAGGAT | ACTCAGCTGCCTGCTTCTTC |  |
| PPARγ | TGACAGCGACTTGGCAAT | TGGGCTTCACATTCAGCA | TATTCTCAGTGGAGACCGCCCAGGT |
| REST | AATTATGTTCAGCATGTTAGAACTCA | GAGGCCACATAACTGCACTG | TGCGTACTCATTCAGGTGAGAAGCCA |
| RPTOR | AAGGGCCCAGAGCAGACT | AGTCGCAGAACCCCGTCT |  |
| SYP | CAGACAGGGAACACATGCAA | AGAGCACCAGGTTCAGGAAG | CGGGACTCAACACCTCGGTGG |

## Supplementary Table 2: Antibodies for Western Blotting

| **Host** | **Target** | **Company** | **Cat. Num.** | **Flourophore** |
| --- | --- | --- | --- | --- |
| Rabbit | LC3A/B | Cell Signaling | 12741 | Unconjugated |
| Mouse | AKT | Cell Signaling | 9272 | Unconjugated |
| Rabbit | p-AKT (Ser473) | Cell Signaling | 4051 | Unconjugated |
| Rabbit | p-AKT (Thr308) | Cell Signaling | 13038 | Unconjugated |
| Rabbit | ULK1 | Cell Signaling | 8054 | Unconjugated |
| Rabbit | p-ULK1 (Ser757) | Cell Signaling | 6888 | Unconjugated |
| Rabbit | p-Ulk1 (Ser317) | Cell Signaling | 89267 | Unconjugated |
| Rabbit | p70S6K | Cell Signaling | 2708 | Unconjugated |
| Rabbit | p-p70S6K | Cell Signaling | 9206 | Unconjugated |
| Mouse | V-ATP5A | Abcam | ab110411 | Unconjugated |
| Mouse | III-UQCRC2 | Abcam | ab110411 | Unconjugated |
| Goat | β-actin | Abcam | ab8229 | Unconjugated |

## Supplementary Table 3: Antibodies

| **Host** | **Target** | **Company** | **Cat. Num.** | **Flourophore** |
| --- | --- | --- | --- | --- |
| Horse | Rabbit | Vector Labs | DI-1094 | DyLight 594 |
| Horse | Mouse | Vector Labs | DI-2594 | DyLight 594 |
| Goat | Rat | Abcam | ab98422 | DyLight 594 |
| Donkey | Goat | Invitrogen | A-11058 | Alexa Fluor 594 |
| Donkey | Goat | LI-COR | 926-68074 | IRDye 680RD |
| Donkey | Rabbit | LI-COR | 926-32213 | IRDye 800CW |
| Donkey | Mouse | LI-COR | 926-49012 | VRDye 490 |

## Supplementary Table 4: Antibodies for Confocal Microscopy

| **Host** | **Target** | **Company** | **Cat. Num.** | **Flourophore** |
| --- | --- | --- | --- | --- |
| Mouse | aSMA | Dako | M0851 | Unconjugated |
| Mouse | REST | Abcam | ab211537 | Unconjugated |
| Rabbit | Synaptophysin | Abcam | ab32127 | Unconjugated |
| Rabbit | COL1A1 | Cell Signaling | 72026 | Unconjugated |
